# Photosynthetic efficiency, desiccation tolerance and ultrastructure in two phylogenetically distinct strains of alpine *Zygnema* sp. (Zygnematophyceae, Streptophyta): role of pre-akinete formation

**DOI:** 10.1007/s00709-014-0703-3

**Published:** 2014-10-01

**Authors:** K. Herburger, L. A. Lewis, A. Holzinger

**Affiliations:** 1 Institute of Botany, Functional Plant Biology, University of Innsbruck, Sternwartestrasse 15, 6020 Innsbruck, Austria; 2 Department of Ecology and Evolutionary Biology, University of Connecticut, Storrs, CT 06269-3043 USA

**Keywords:** Alps, Colonization of land, Hydroterrestrial green algae, *rbc*L phylogeny, rETR, Temperature, Transmission electron microscopy

## Abstract

Two newly isolated strains of green algae from alpine regions were compared physiologically at different culture ages (1, 6, 9 and 15 months). The strains of *Zygnema* sp. were from different altitudes (‘Saalach’ (S), 440 m above sea level (a.s.l.), SAG 2419 and ‘Elmau-Alm’ (E-A), 1,500 m a.s.l., SAG 2418). Phylogenetic analysis of *rbc*L sequences grouped the strains into different major subclades of the genus. The mean diameters of the cells were 23.2 μm (*Zygnema* S) and 18.7 μm (*Zygnema* E-A) but were reduced significantly with culture age. The photophysiological response between the strains differed significantly; *Zygnema* S had a maximal relative electron transport rate (*rETR*_max_) of 103.4 μmol electrons m^−2^ s^−1^, *Zygnema* E-A only 61.7 μmol electrons m^−2^ s^−1^, and decreased significantly with culture age. Both strains showed a low-light adaption and the absence of strong photoinhibition up to 2,000 μmol photons m^−2^ s^−1^. Photosynthetic oxygen production showed similar results (*P*_max_ *Zygnema* S, 527.2 μmol O_2_ h^−1^ mg^−1^ chlorophyll (chl.) *a*, *Zygnema* E-A, 390.7 μmol O_2_ h^−1^ mg^−1^ chl. *a*); the temperature optimum was at 35 °C for *Zygnema* S and 30 °C for *Zygnema* E-A. Increasing culture age moreover leads to the formation of pre-akinetes, which accumulate storage products as revealed by light and transmission electron microscopy. Desiccation at 84 % relative air humidity (RH) lead to a reduction of the effective quantum yield of photosystem II (PSII) (Δ*Fv*/Fm′) to zero between 90 to 120 min (*Zygnema* S) and between 30 to 60 min (*Zygnema* E-A), depending on the culture age. A partial recovery of Δ*Fv*/Fm′ was only observed in older cultures. We conclude that pre-akinetes are crucial for the aeroterrestrial lifestyle of *Zygnema*.

## Introduction

*Zygnema* (Zygnematophyceae), a streptophyte green alga, occurs in freshwater (Ettl and Gärtner 1995; Hawes 1989) and hydroterrestrial habitats (Ettl and Gärtner 1995; Elster and Benson 2004; Holzinger et al. 2009), including shallow puddles, streams and well-hydrated soils in alpine, Arctic and Antarctic regions (Davey 1991; Holzinger et al. 2009; Stancheva et al. 2012; Kaplan et al. 2013; Pichrtová et al. 2013, 2014). In addition to its widespread occurrence (Stancheva et al. 2012; Hall et al. 2008) *Zygnema* is also of considerable interest in an evolutionary context. It is commonly accepted that Zygnematophyceae (Timme et al. 2012) or a taxon comprising Zygnematophyceae and Coleochaetophyceae (Wodniok et al. 2011; Friedl and Rybalka 2012; Leliaert et al. 2012) is sister to land plants (embryophytes). The successful colonization of terrestrial habitats in the Ordovician period (~460 MY ago) by streptophyte green algae might have been enabled by physiological pre-adaptation to terrestrial habitats as a result of their freshwater lifestyle (Becker and Marin 2009). Today, many representatives of streptophyte algae occur in aeroterrestrial habitats (Lewis and McCourt 2004; Lewis 2007), where they have to cope with diurnal temperature fluctuations including frost events, water scarcity, lack of nutrients and high light and UV intensities (Holzinger et al. 2009; Holzinger and Karsten 2013; Pichrtová et al. 2013, 2014; Aigner et al. 2013; Stancheva et al. 2014).

The ultrastructure (McLean and Pessoney 1970, 1971; Bakker and Lokhorst 1987; Holzinger et al. 2009; Kaplan et al. 2013; Pichrtová et al. 2013) and ecophysiological performance after different stress treatments (Holzinger et al. 2009; Kaplan et al. 2013; Vilumbrales et al. 2013; Pichrtová et al. 2014) are well described in the genus *Zygnema*. Holzinger et al. (2009) did not detect significant changes to cellular ultrastructure or photosynthetic performance after experimentally increasing UV to photosynthetic active radiation (PAR) treatment of field-collected arctic strains of *Zygnema* sp. The occurrence of phenolic compounds after exposure to UV stress was investigated recently (Pichrtová et al. 2013), which demonstrated a significant increase in these compounds. Moreover, the aeroterrestrial lifestyle goes along with dehydration, which causes structural and biochemical damage in the cells, including disintegration of organelles or an increase of cellular ionic concentrations (recently reviewed by Holzinger and Karsten 2013). Water stress leads to suppression of photosynthesis and respiration (e.g. Gray et al. 2007; Karsten et al. 2010, 2014; Holzinger et al. 2011; Karsten and Holzinger 2012; Aigner et al. 2013), for example, caused by decreasing rates of repair of D1 protein in photosystem II (PSII) resulting from direct inactivation of the translation machinery (Takahashi and Murata 2008). This leads to interrupted carbon fixation and the creation of reactive oxygen species (ROS), which can cause severe cellular damages by membrane lipid peroxidation and hydroxylation of DNA (Kranner and Birtic 2005). Water loss was investigated by plasmolysis experiments in cultured *Zygnema*, leading to a severe decrease in photosynthetic performance (Kaplan et al. 2013). Also, the effects of plasmolysis were tested recently in arctic field samples of *Zygnema* (Pichrtová et al. 2014). 

These studies mainly focused on strains obtained from Arctic or Antarctic habitats. In contrast, knowledge about the response to stress of *Zygnema* occurring in alpine regions is missing so far. Compared to polar regions, alpine habitats exhibit higher annual mean temperatures (Billings 1973; Körner 2003; Elster and Benson 2004). This is linked to a longer snow-free season and higher soil temperature in the subalpine layer, resulting in a longer growing season (Billings 1973). In contrast, in polar regions, snow melt and thawing of the surface soils take almost half of the sun season (Billings 1973). Furthermore, polar plant life is influenced by polar day and polar night (Billings 1973).

The Alps are considered to be particularly susceptible to climate change (Beniston et al. 1996; Lischke et al. 1998), and it has been shown that the past decade (2000–2009) has been the warmest described by instrumental measurements so far (Zhao and Running 2010). As an effect on terrestrial algae by increased temperatures is expected (Holzinger and Karsten 2013), knowledge of temperature requirements and resistance against water stress in alpine green algae will contribute to a better understanding of changes in terrestrial ecosystems. Isolating algae from different altitudes within the Alps enables to investigate organisms that have to deal with different environmental conditions (Billings 1973; Körner 2007). 

So far, most physiological studies using *Zygnema* sp. emphasized on long-term survival in response to freezing or desiccation, as these algae were obtained from polar habitats (e.g. Hawes 1990; Pichrtová et al. 2014). In these habitats, the availability of liquid water is mostly restricted to the spring melt, exposing the algae to prolonged desiccation during other parts of the year (Holzinger et al. 2009; Kaplan et al. 2013; Pichrtová et al. 2014). *Zygnema* does not possess constitutive desiccation tolerance (McLean and Pessoney 1971); thus, it can be assumed that either acclimation to environmental conditions or the formation of resistant cells enables survival (Pichrtová et al. 2014). The formation of pre-akinetes and akinetes derived from vegetative cells is common in *Zygnema* (Fritsch 1945; Evans 1958; McLean and Pessoney 1971; Kaplan et al. 2013; Pichrtová et al. 2013) and other green algae (Coleman 1983). According to the previously published literature, we use the term ‘pre-akinetes’ for storage-compound-filled cells that are still connected in filaments, whereas single-celled ‘akinetes’ have not been observed in this study. It is already stated by McLean and Pessoney (1971) that the usage of this terminology differs from the taxonomic literature (Transeau 1951). Stancheva et al. (2012) use the term akinete in this sense, depending on the phylogenetic position; akinetes with stained or colourless mesospore have been described in *Zygnema* sp.

These resistant cells (‘pre-akinetes’) develop from vegetative cells and are likely to be a key factor in surviving unfavourable environmental conditions, nutrient and water scarcity or prolonged low temperatures (Pichrtová et al. 2014). Also, senescent or growth-limited vegetative cells in the stationary growth phase subsequently produce akinetes (McLean and Pessoney 1971). This transition includes cell wall thickening (Evans 1958) and extensive accumulation of starch as wells as lipid bodies in the cytoplasm, accompanied by chloroplasts losing their characteristic stellate shape (McLean and Pessoney 1971).

The basis of the thick cell walls of pre-akinetes in green algae is formed by the original cell wall, followed by incorporation of additional cell wall layers (Coleman 1983; Fuller 2013). It is reported that akinetes of *Zygnema* can survive long-term desiccation, whereas even short-term desiccation is lethal to cells lacking akinete morphology (McLean and Pessoney 1971). Investigating the physiological performance of pre-akinetes is of considerable interest as naturally occurring populations of *Zygnema* sp. frequently form pre-akinetes (Pichrtová et al. 2014). In the present study, we investigated two newly isolated strains of *Zygnema* obtained from alpine habitats in Austria. The strains were characterized according their (1) phylogenetic position, (2) structure and ultrastructure and (3) photosynthetic performance. In addition, the response of cells to (4) desiccation stress was investigated. As older cultures contain predominantly pre-akinetes, we also investigated if these cells differ in their physiological performance compared to vegetative cells, thereby contributing to desiccation tolerance.

## Material and methods

### Strain origin and culture conditions

*Zygnema* sp. ‘Saalach’ (SAG 2419) and *Zygnema* sp. ‘Elmau-Alm’ (SAG 2418) were isolated from hydroterrestrial habitats. *Zygnema* sp. ‘Saalach’ was obtained from the sandy littoral zone of the river Saalach (47° 47′ 8.70′′ N, 12° 56′ 42.66′′ E; 440 m above sea level (a.s.l.)) near Salzburg (Salzburg, Austria). *Zygnema* sp. ‘Elmau-Alm’ was collected from a sun-exposed shallow puddle in the catchment area of a subalpine pasture called ‘Elmau-Alm’ (47° 28′ 52.30″ N, 13° 14′ 48.85″ E, 1,500 m a.s.l.) near Werfenweng (Salzburg, Austria). Both strains were submitted to the Culture Collection of Algae of the Georg-August-Universität Göttingen and assigned strain numbers (see above). Samples were purified and established into unialgal cultures. Algae were cultivated on 1.5 % agar plates or in 250–500-mL Erlenmeyer flasks containing Bold’s Basal Medium (BBM; Bischoff and Bild 1963), respectively. Agar plates were sealed with Parafilm® to keep the moisture level constant during long-term cultivation. All algae grew in a dark/light regime of 16:8 h adjusted in a thermostat (Percival PGC 6L, Percival Scientific, Perry, GA, USA) at 20 °C and ~33 μmol photons m^−2^ s^−1^ in the light period. In the dark, temperature was reduced to 14.5 °C. Light was provided by Osram Daylight Lumilux Cool White lamps (L36W⁄840; Osram. Munich, Germany).

### DNA sequencing and phylogenetic analysis

DNA was isolated from the two *Zygnema* strains using the PowerPlant DNA Isolation Kit (Mo Bio Laboratories, Inc., Carlsbad, CA, USA). PCR amplification of the ribulose-bisphosphate carboxylase long chain (*rbc*L) gene was performed using primers M28F, M1161R or M1390R (McManus and Lewis 2011; Pichrtová et al. 2013). Sequencing with the PCR primers plus primers 443F and 1263R (Pichrtová et al. 2013) yielded at least three reads to produce consensus sequences. PCR and sequencing conditions followed Kaplan et al. (2012). Products of cycle sequencing were run on an ABI 3100 DNA Sequencer™ (Applied Biosystems, Foster City, CA, USA), with individual reads compiled into contigs in Sequencher 4.5 (Gene Codes Inc, Ann Arbor, MI, USA) and edited manually to resolve any ambiguity. *rbc*L sequences from the study strains of *Zygnema* were compared to the NCBI database through BLAST searches (Altschul et al. 1990), and the resulting top matches of those were used to produce an alignment. Inclusion of additional strains in the alignment was also informed by the most recent *Zygnema* phylogeny (Stancheva et al. 2012). 

The model sequence evolution to be used in the maximum likelihood (ML) and Bayesian analyses was selected under the Akaike information criterion (AIC) as general time reversible (GTR) + I + gamma and TrN + I + gamma under the Bayesian information criterion (BIC). Analyses were run under both models, but as the resulting trees were very similar, only results from the first are shown. ML analyses with bootstrapping (200 replicates) were performed in PAUP* (Swofford 2002). The GTR + I model parameter values were set during the search based on a pilot analysis: *R*_A-C_ = 1.7130851, *R*_A-G_ = 6.6467111, *R*_A-T_ = 2.9992596, *R*_C-G_ = 1.4440773, *R*_C-T_ = 14.5219168, *R*_G-T_ = 1, pinvar = 0.591022 and gamma shape = 0.906714 (four rate categories). The Bayesian analyses were done in MrBayes 3.2.1 (Huelsenbeck and Ronquist 2001; Ronquist and Huelsenbeck 2003). Two independent Bayesian analyses were run for 4.1 × 10^6^ generations with one cold plus three heated chains, with a subsample frequency of 1,000. Convergence was determined, and trees from the initial 105 generations were discarded as burn in before producing the majority-rule consensus tree. Lastly, we performed parsimony bootstrap analysis (1,000 replicates) using PAUP* with TBR branch swapping.

### Light microscopy

Algal cells of cultures of different age were taken from Erlenmeyer flasks (1–2-month-old cultures) or agar plates (~6, ~9 and ~15-month-old cultures) to determine their cell dimensions (cell width and cell length) with a minimum of 20 cells by using a Zeiss Axiovert 200M microscope, equipped with a 63 × 1.4 NA objective lens. Cell dimensions of algal cells from 1–2-month-old liquid culture were equal to cells from agar plates. Long-time cultivation (>~6 months) was solely conducted on agar plates. Images were generated by using differential interference contrast (DIC) and captured with an Axiocam MRc5 camera and Zeiss Axiovision software. Images were further processed with Adobe Photoshop (CS5) software version 12.1 (Adobe Systems, San Jose, CA, USA).

### Transmission electron microscopy

For transmission electron microscopy, samples of *Zygnema* sp. ‘Saalach’ and *Zygnema* sp. ‘Elmau-Alm’ of different culture age (1 and 6 months) were taken from agar plates. The 6-month-old culture was chosen to ensure that pre-akinetes were present in the samples. Algal filaments were prepared according to Holzinger et al. (2009). Briefly, desiccated algal filaments were fixed for 1.5 h in 20-mM cacodylate buffer (pH = 7.0) containing 2.5 % glutaraldehyde and postfixed in 1 % osmium tetroxide for ~16 h at 4.6 °C. Probes were rinsed, dehydrated by transferring to increasing ethanol concentrations and propylenoxid and embedded in Spurr’s resin modified after Ellis (2006). After preparing ultrathin sections by using a Reichert Ultracut (Leica Microsystems, Wetzlar, Germany), probes were counterstained with 2 % uranyl acetate and Reynold’s lead citrate and examined with a Zeiss Libra 120 TEM (80 kV) connected to a ProScan 2 k SSCCD camera, controlled with OSIS iTEM software. Images were further processed with Adobe Photoshop (CS5) software. 

### Photosynthetic oxygen production and respiration measurements by an oxygen optode

Photosynthetic oxygen production rates as a function of increasing photon fluence rates (PI curves) and respiratory oxygen consumption in the dark were recorded by using a Presens Fibox 3 oxygen optode (Presens, Regensburg, Germany). According to Remias et al. (2010), the oxygen sensor was attached to a 3 mL thermostatic acrylic chamber (type DW1, Hansatech Instruments, Norfolk, UK), placed on a magnetic stirrer. The chamber was filled with 2.8-mL algal suspension taken from 1–2 month-old liquid cultures as there was no difference in the photosynthetic performance of young algae compared to same-aged algae cultivated on agarose media. Physiological differences between older cultures (>~6 months) in liquid and hard media were not tested. Additionally, algal suspension was enriched with 0.2-mL NaHCO_3_ stock solution, resulting in a final concentration of 2 mM NaHCO_3_ to counteract carbon shortage during measurement. The algae were exposed to nine photon fluence rates (0–500 μmol photons m^−2^ s^−1^ PAR; for further details see Karsten and Holzinger 2012). Directly before the onset of increasing photon fluence rates (each stage 10 min) as well as directly after offset of the final light level, respiration was measured for 6 min by darkening the chamber. Respiration (R) was determined by calculating the mean of these two values. To ensure constant temperatures in the measuring chamber a Thermo Haake K20 refrigerated circulator (Thermo Fisher Scientific Inc., Waltham, MA, USA) was connected to the system. The O_2_ production per photon fluence rates and per time was normalized to the content of chlorophyll *a* per sample. After each PI curve measurement, cell suspension was collected onto an Ø 47-mm Whatman GF/F glass microfibre filter (Whatman, Dassel, Germany) by using a glass pipette. Chlorophyll *a* was extracted with 3 mL dimethyl formamide (Sigma-Aldrich, Steinheim, Germany) and quantified photometrically (Porra et al. 1989). Calculated PI curve data were mathematically described by the fitting model of Webb et al. (1974; without photoinhibition) or by using the model of Walsby (1997) (with photoinhibition), which enabled to calculate four characteristic parameters: *α*, positive slope at limiting photon fluence rates (μmol O_2_ h^−1^ mg^−1^ chlorophyll (chl.) *a* (μmol photons^−1^ m^−2^ s^−1^)^−1^); *I*_c_, light compensation point (μmol photons m^−2^ s^−1^); *I*_k_, initial value of light-saturated photosynthesis (μmol photons m^−2^ s^−1^) and *P*_max_, maximum photosynthetic oxygen production in the light saturation range (μmol O_2_ h^−1^ mg^−1^ chl. *a*). 

### Measurements of relative electron transport rates

A pulse-amplitude modulated fluorimeter (PAM 2500, Heinz Walz GmbH, Effeltrich, Germany) was used to determine the relative electron transport rates (rETRs) in response to increasing photon flux densities. The parameters were evaluated and calculated according to Kromkamp and Forster (2003): rETR = Δ*F*/*Fm*′ PFD, where Δ*F*/*Fm*′ = the effective quantum yield of PSII and PFD = photon flux density. This was always performed for algal cultures of four different ages (1–2, ~6, ~9 and ~15 months) taken from agar plates. Algal filaments (c. 1–1.5 mg chlorophyll *a* L^−1^, determined by using dimethyl formamide, see above) were added to a KS-2500 suspension cuvette (Heinz Walz GmbH, Effeltrich, Germany) containing 400 μL of standard growth medium. The PAM light probe was connected to the cuvette to expose algal filaments to 17 light steps (each 30 s) ranging from 2 to 2,015 μmol photons m^−2^ s^−1^ PAR. Actinic light was provided by a red LED (630 nm). Each light step was followed by a saturation pulse to determine Δ*F*/*Fm*′. The light response curve was fitted by the models of Webb et al. (1974) or Walsby (1997) depending on either photoinhibition occurred or not. Three of the photosynthesis parameters *rETR*_max_ (maximum electron transport rate; μmol electrons m^−2^ s^−1^), *α* (electrons photon^−1^) and *I*_k_ (μmol photons m^−2^ s^−1^) were derived.

### Temperature requirements of photosynthesis and respiration measurements by using an oxygen optode

A Thermo Haake K20 refrigerated circulator (Thermo Fisher Scientific Inc., Waltham, MA, USA) was connected to the chamber to examine the effect of changing temperatures on photosynthetic oxygen production and respiratory consumption, respectively. According to Karsten and Holzinger 2012, algae from 1–2-month-old liquid cultures were exposed to nine rising temperature steps (5 to 45 °C in 5 °C increments). Analogous to light measurements (see above), the O_2_ consumption and production per time unit were referenced to the total amount of chlorophyll *a* per sample. Additionally, the gross photosynthesis to respiration (P/R) ratios for each temperature were calculated.

### Dehydration experiments and determination of the effective quantum yield

For monitoring changes in effective quantum yields of photosystem II (Δ*F*/*Fm*′; PSII) during desiccation and subsequent rehydration, a standardized set-up was used as described in Karsten et al. 2014. Briefly, algal filaments (c. 1–1.5 mg chlorophyll *a* L^−1^) from agar plates were transferred to Whatman GF/F glass fibre filters that were moistened with 20 μL of BBM (four replicates). This was performed independently with algal cultures of three different ages (1, 6 and 15 months). Prepared filters were adjusted on perforated metal grids in a transparent 200-mL polystyrol box (*d* = 12 cm), which was filled with 150 mL of saturated KCl solution (Merck, Darmstadt, Germany) for setting relative air humidity (RH) inside the chamber to ~84 % (Greenspan 1977). Additionally, RH was recorded by using a PCEMSR145S-TH mini data logger (PCE Instruments, Meschede, Germany). The boxes were placed under a halogen lamp (40 μmol photons m^−2^ s^−1^ PAR) at ambient room temperature (23 ± 1 °C). A PAM 2500 was used to determine Δ*F*/*Fm*′ of PSII (Genty et al. 1989; Schreiber and Bilger 1993) continuously during dehydration (60–120 min), whereas the PAM light probe was adjusted outside the sustained sealed chamber with a 2-mm distance to the cover lid. This resulted in a total distance from the PAM light probe to the algal sample of constant 12 mm. Subsequently after dehydration, filters were rehydrated by adding 20 μL of the standard growth medium to each algal sample and transferred to a polystyrol box containing 100 mL tap water to create a higher RH (~96 %). Measuring recovery of Δ*F*/*Fm*′ was performed analogously as described above. 

### Statistical evaluation of the data

All experiments determining rETR, Δ*F*/*Fm*′ and oxygen measurements were carried out with three independent replicates. Desiccation experiments determining Δ*F*/*Fm*′ were carried in four independent replicates. Data are represented by their means and standard deviation. Analysis of temperature and desiccation effects of photosynthesis (oxygen production, Δ*F*/*Fm*′) as well as comparison of cell lengths and widths (*n* = 20) were performed by one-way analysis of variance (ANOVA), followed by Tukey’s post hoc test (*p* < 0.05) to find homogeneous subgroups of significantly different means. Comparison of characteristic photosynthetic parameters (for rETR *α*, *I*_k_, *rETR*_max_; for PI curves *α*, *I*_c_, *I*_k_, *R*, *P*_max_) was performed by a standard two-sample *t* test (*p* < 0.001). Analyses were carried out in Origin 8.5 (OriginLab Corporation, Northampton, MA, USA).

## Results

### Molecular characterization

**Fig. 1:**
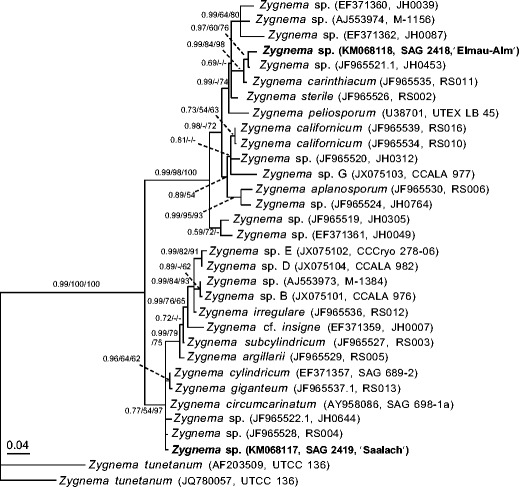
Unrooted maximum likelihood phylogenetic tree (lnL = −4,175.608) showing the placement of the two focal *Zygnema* strains used in this study (*boldface font*) in the context of a *Zygnema* phylogeny (Stancheva et al. 2012). Taxon labels include the corresponding GenBank accession numbers and strain numbers in *parentheses*. Branch support values are indicated (Bayesian posterior probability/ML bootstrap/parsimony bootstrap) for values over 0.5/50/50. *Scale bar* = number of expected substitutions/site

*rbc*L sequences of the two new *Zygnema* strains were deposited in GenBank under accession numbers KM068117 (*Zygnema* sp., ‘Saalach’, SAG 2419), and KM068118 (*Zygnema* sp. ‘Elmau-Alm’, SAG 2418). The sequences obtained were 1,243 (SAG 2419, ‘Saalach’) and 1,120 (SAG 2418, ‘Elmau-Alm’) nucleotides in length. The *rbc*L alignment includes 32 *Zygnema* taxa in total, 1,384 nucleotide positions (1,354 after exclusion of 30 nucleotides from the 5′-most end that was missing data in most sequences), with 1,102 constant and 173 parsimony informative sites. Results of the phylogenetic analyses demonstrate that the two focal *Zygnema* strains are not closely related (Fig. 1). Instead, *Zygnema* sp. ‘Elmau-Alm’ is closest to strain *Zygnema* sp. JH0453 and *Zygnema carinthiacum* Beck-Mannagetta. *Zygnema* sp. ‘Saalach’ is related to several species including *Zygnema circumcarinatum* Czurda and strains isolated by J. Hall (JH0644) and R. Stancheva (RS004). The distinction of *Zygnema* sp. ‘Saalach’ and *Zygnema* sp. ‘Elmau-Alm’ is supported strongly by the Bayesian, ML and parsimony bootstrap analyses.

### Light microscopy

**Fig. 2:**
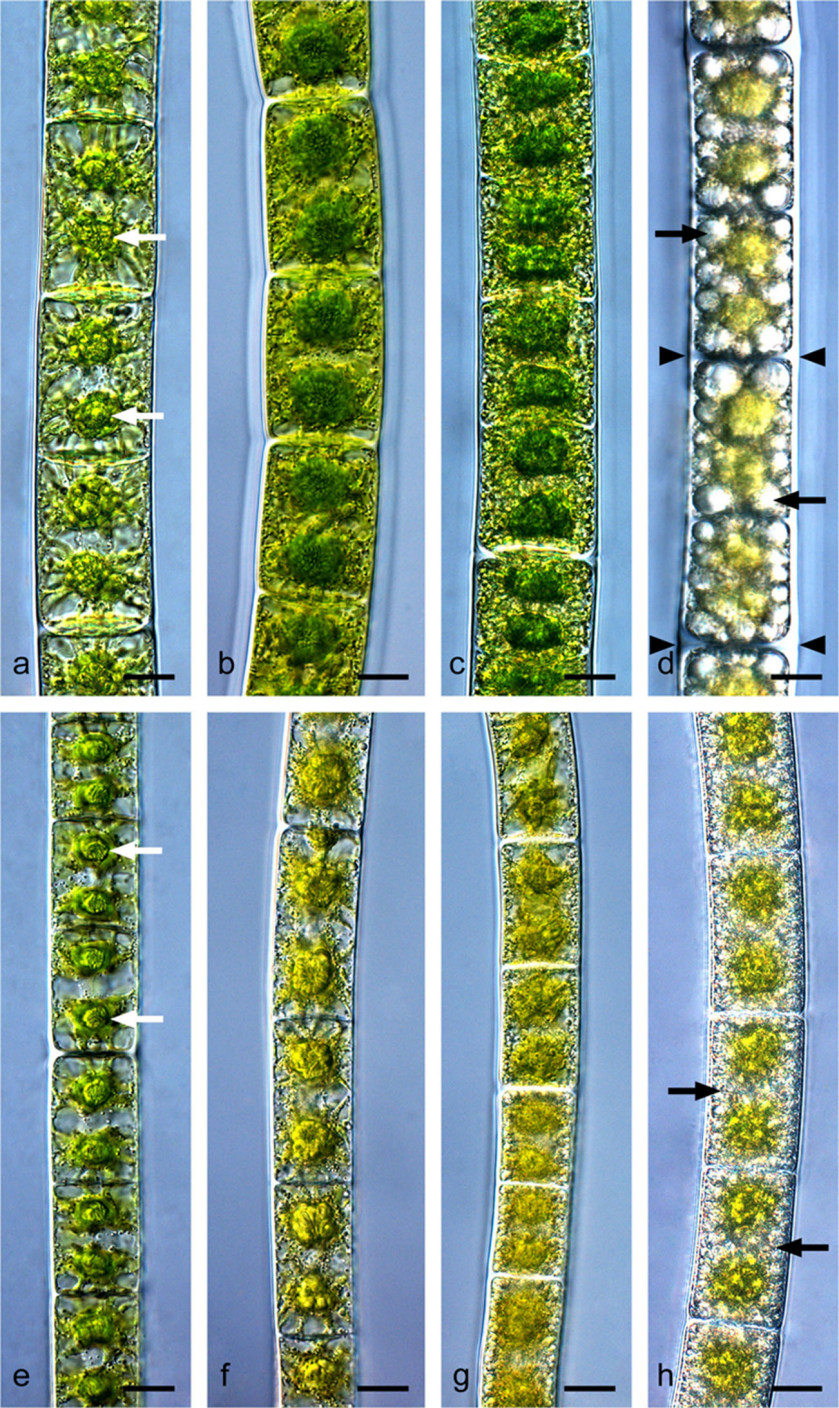
Morphology of **a**–**d** *Zygnema* sp. ‘Saalach’ and **e**–**h** *Zygnema* sp. ‘Elmau-Alm’ from cultures of different age (**a**, **e** 1 month; **b**, **f** 6 months; **c**, **g** 9 months; **d**, **h** 15 months). **a** Young vegetative cells, pyrenoids are marked with *arrows*, (**b**) beginning pre-akinete formation, **c** pre-akinetes, **d** pre-akinetes of old cultures, accumulation of starch grains and lipid bodies (*arrow*) and thickened cell walls (*arrowheads*), **e** young vegetative cells, pyrenoids marked with *arrows*, **f** older vegetative cells, **g** pre-akinetes, **h** pre-akinetes filled with storage compounds (*arrows*). *Bars* 10 μm

Both strains formed uniserate filaments. Cells taken from 1–2-month-old liquid cultures of *Zygnema* sp. ‘Saalach’ had a diameter of 23.20 ± 1.26 μm; their cell length varied and had a mean value of 31.94 ± 6.85 μm (Table 1). *Zygnema* sp. ‘Elmau-Alm’ cells of the same age had a diameter of 18.65 ± 0.83 μm and a length of 27.96 ± 3.14 μm (Table 1). Again, the diameter was more homogenous than the length. Cultures (1–2 month old) from both ‘Saalach’ and ‘Elmau-Alm’ had vacuolated cells, and each cell contained two star-shaped chloroplasts, forming narrow lobes that were protruding towards the cell periphery (Fig. 2a, e). Each chloroplast contained a centrally located pyrenoid surrounded by starch grains. Starch or lipid accumulation within the cytoplasm was not observed in these young cells.

**Table 1:** Lengths and widths (in μm) of cells of the two *Zygnema* strains obtained from cultures of different age (*n* = 20 ± SD)

	1–2 months		6 months		9 months		15 months	
	Length	Width	Length	Width	Length	Width	Length	Width
*Zygnema* sp. ‘Saalach’	31.94 ± 6.85A	23.17 ± 1.26a	27.31 ± 5.93A	24.97 ± 0.79b	31.13 ± 4.31A	22.83 ± 0.81c	33.97 ± 6.60B	21.58 ± 1.24d
*Zygnema* sp. ‘Elmau-Alm’	27.96 ± 3.14A*	18.65 ± 0.83a*	24.24 ± 5.71A	17.81 ± 0.64b*	31.61 ± 7.19AB	17.04 ± 0.60c*	38.68 ± 5.53C*	16.90 ± 0.57d*

Significant differences between the dimensions of different aged cultures of the same strain are indicated by capital letters (length) and small letters (width). Cell dimensions of different strains obtained from cultures of the same age were compared, and when ‘Elmau-Alm’ was significantly different from ‘Saalach’, it is marked with an asterisk (*p* < 0.05). Data were analysed by one-way ANOVA followed by Tukey’s post hoc test

Cells from older cultures (6 to 9 months) underwent a transformation to pre-akinetes (Fig. 2b, c, f, g). They exhibited higher amounts of storage compounds compared to 1–2-month-old cultures, likely representing starch grains or lipid bodies, especially in the outer part of cytoplasm (Fig. 2c, g). Additionally, decreasing vacuolization, shape loss of the chloroplasts, pyrenoids that were less distinct, and increasing cell wall thickness were correlated with increasing age (Fig. 2). Furthermore, cells from older cultures varied in their dimensions (Table 1). Compared to the youngest cultures, the width of cells taken from 6-month- old cultures of *Zygnema* sp. ‘Saalach’ was significantly higher; however, it decreased significantly after 9 months, reaching a minimum in the 15-month-old culture (*p* < 0.05; Table 1). The cell width of *Zygnema* sp. ‘Elmau-Alm’ decreased continuously with age (*p* < 0.05; Table 1). In contrast, the cell length of *Zygnema* sp. was significantly higher in the 15-month- old culture, while in *Zygnema* sp. ‘Elmau-Alm’, it increased significantly in the 9-month-old culture, reaching its maximum after 15 months (*p* < 0.05; Table 1). Comparing the cell width of the two *Zygnema* strains showed that cells of *Zygnema* sp. ‘Saalach’ were wider in every case (*p* < 0.05; Table 1). In contrast, significant differences (*p* < 0.05) between the two strains occurred only in the youngest and oldest culture (Table 1). Cultures kept for more than 1 year predominantly contained pre-akinetes characterized by conspicuous amounts of storage products and thicker cell walls compared with previous stages (Fig. 2d, h). However, a separation of akinetes from parent filaments was not observed.

### Transmission electron microscopy

**Fig. 3:**
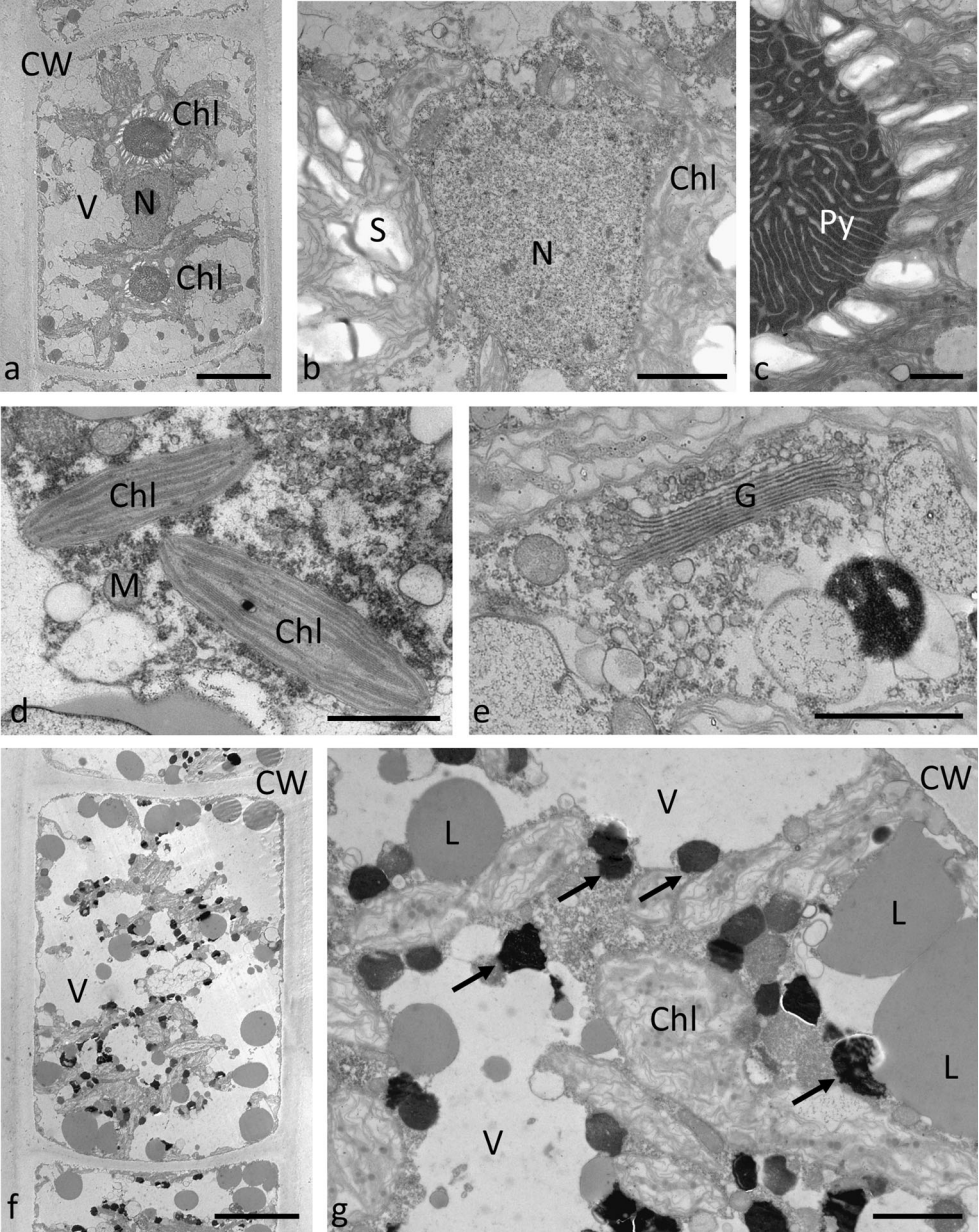
Transmission electron micrographs of *Zygnema* ‘Saalach’ from cultures of different age (**a**–**e** 1 month, **f**–**g** 6 months). **a** Longitudinal section through a young cell showing large vacuoles and chloroplast lobes spreading towards the cell periphery, **b** a central area showing the nucleus and chloroplasts containing large amounts of starch, **c** detail of the pyrenoid with electron-dense matrix surrounded by starch grains, **d** chloroplast lobes, **e** Golgi body with numerous vesicles, **f** longitudinal section though older cells containing numerous lipid bodies and electron-dense bodies, **g** detail of the cytoplasm of older cells containing lipid bodies and electron-dense bodies in close contact to chloroplast lobes. *Bars* **a**, **f** 10 μm; **b**, **g** 2 μm; **c**, **d**, **e** 1 μm

Transmission electron microscopy of *Zygnema* sp. ‘Saalach’ revealed two chloroplasts, each with one pyrenoid surrounded by numerous starch grains (Fig. 3a–c). The nucleus had a central position and was embedded among the chloroplast lobes (Fig. 3b). When young cells were investigated, large vacuoles were observed between the chloroplast lobes (Fig. 3a). The chloroplast lobes were narrow and had a diameter of approx. 0.5 to 1 μm; occasionally, the chloroplast lobes were found to be branched. The thylakoid membranes were parallel arranged and spread throughout the chloroplast (Fig. 3). The cell wall was homogenous and approx. 1 μm thick. Occasionally, an outer mucilage layer of approx. the same diameter was observed. This layer was less electron dense as the cell wall and contained bacteria (not shown). The mitochondria had a diameter of ~0.3–0.5 μm, and cristae were clearly visible (Fig. 3). In contrast to younger cells, cells taken from 6-month-old cultures grown on agar plates contained numerous lipid droplets and electron-dense granules (Fig. 3f–g). The lipid droplets had a smooth appearance, medium electron density and a diameter of up to 1–2 μm. Electron-dense particles with a diameter of up to 600 nm were irregular in shape and sometimes condensed, usually found in close contact to chloroplasts (Fig. 3g).

**Fig. 4:**
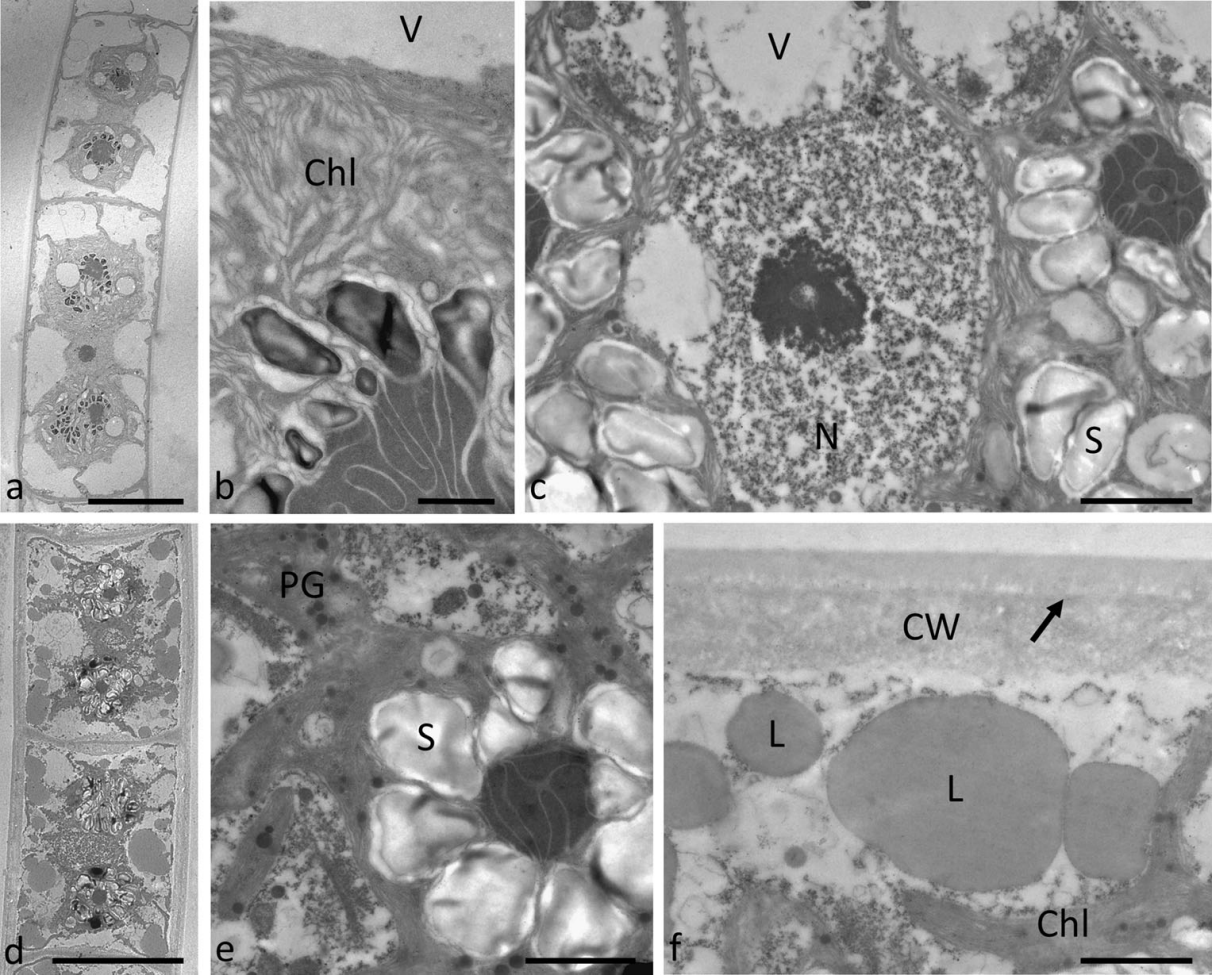
Transmission electron micrographs of *Zygnema* ‘Elmau-Alm’ from cultures of different age (**a**–**c** 1 month, **d**–**f** 6 months). **a** Longitudinal section through two young cells containing large vacuoles, a central nucleus and two chloroplasts with pyrenoids, **b** detail of the chloroplast with thylakoid membranes, and a pyrenoid surrounded by starch grains, **c** central area with nucleus, chloroplasts densely packed with starch grains, **d** longitudinal section through older cells containing large lipid droplets and smaller vacuoles, **e** detail of a chloroplast from an older cell containing a pyrenoid with starch grains and plastoglobules, **f** lipid droplets in the cell periphery of an older cell, the cell wall was composed of two layers. *Bars* **a d** 20 μm; **c**, **e**, **f** 2 μm; **b** 1 μm

The ultrastructure of *Zygnema* sp. ‘Elmau-Alm’ also showed a central nucleus (Fig. 4a, c) and two chloroplasts with narrow lobes protruding to the cell periphery (Fig. 4a). Pyrenoids had a central electron-dense stroma surrounded by individual starch grains with a diameter of up to 1 μm; occasionally, two or more pyrenoids per chloroplast were observed (Fig. 4c). Other organelles such as Golgi bodies and mitochondria were similar as observed above. Older cells contained large lipid droplets with a diameter of up to 1–2 μm (Fig. 4d, f). Their cell walls were thicker, and two layers were clearly visible (Fig. 4d, f) The chloroplasts still contained pyrenoids and starch grains, and plastoglobules were evident (Fig. 4e).

### Light requirements for photosynthesis

#### Relative electron transport rates

**Fig. 5:**
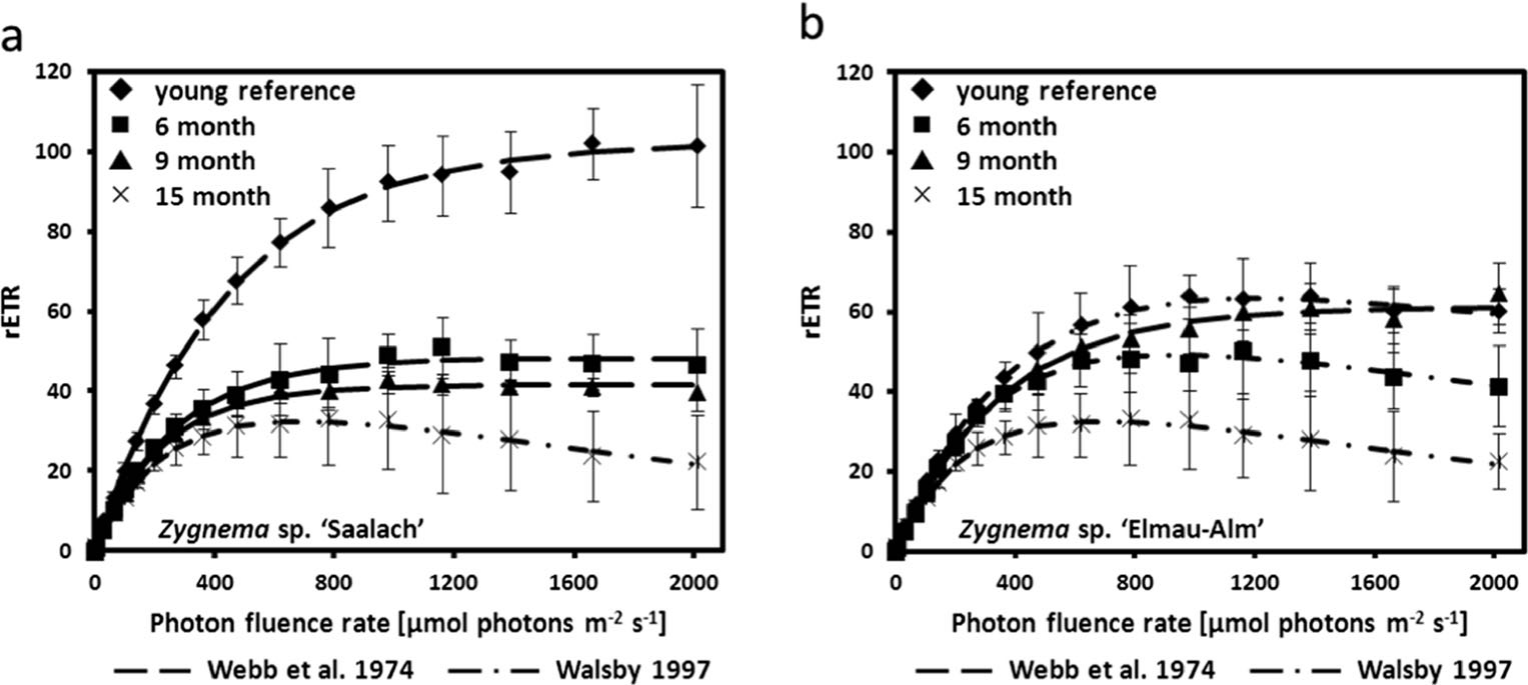
Relative electron transport rates (rETRs, μmol electrons m^−2^ s^−1^) as a function of increasing light intensities up to 2,015 μmol photons m^−2^ s^−1^ in two *Zygnema* strains of always four different age (*n* = 3, mean value ± SD). rETR curves were determined by using the fitting models of Walsby 1997 (*dot-dashed line*) or Webb et al. 1974 (*dashed line*) depending on either photoinhibition occurred or not. **a** *Zygnema* sp. ‘Saalach’, **b** *Zygnema* sp. ‘Elmau-Alm’. Characteristic parameters of the most recent rETR curves are listed in Table 2

**Fig. 6:**
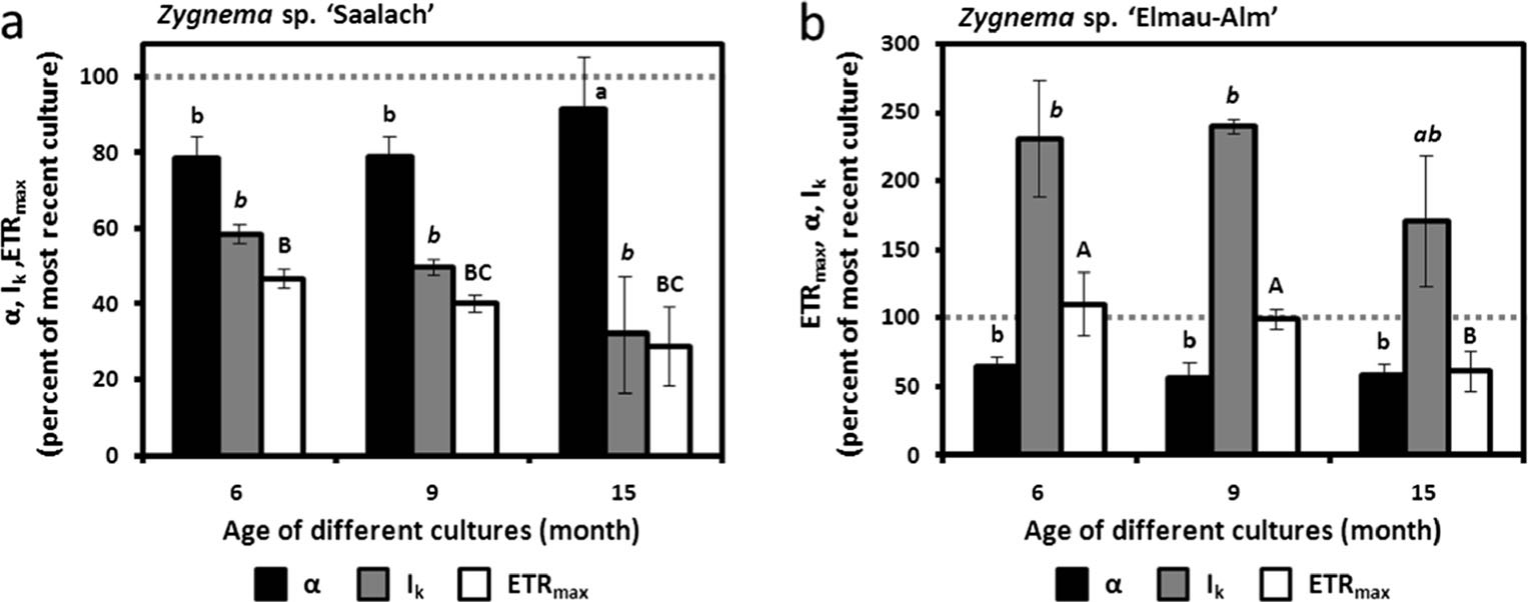
Comparison of photosynthesis parameters derived from rETR curves in **a** *Zygnema* sp. ‘Saalach’ and **b** *Zygnema* sp. ‘Elmau-Alm’, determined by using the fitting models of Walsby 1997 or Webb et al. 1974 (depending on whether or not photoinhibition occurred). Values from the youngest cultures are listed in Table 3. They were followed by standardization to 100 % for better comparison (indicated as a *grey dashed line*). Significances between the treatments are indicated by *small letters* (*α*), *cursive letters* (*I*_k_) and *capital letters* (*rETR*_max_). They were determined by one-way ANOVA (*p* < 0.05) followed by Tukey’s post hoc test

Measured rETR in response to increasing photon fluence rates up to 2,015 μmol photons m^−2^ s^−1^ showed variation between the two *Zygnema* strains as well as between cultures of different age (Fig. 5). Younger cultures of *Zygnema* sp. ‘Saalach’ (1–2 months) exhibited the highest measured *rETR*_max_ value (103.43 ± 13.22 μmol electrons m^−2^ s^−1^; Fig. 5a), while *Zygnema* sp. ‘Elmau-Alm’ taken from a culture with analogous age amounted to 61.74 μmol electrons m^−2^ s^−1^ (Table 2, Fig. 5b). The *α* value in *Zygnema* sp. ‘Saalach’ was significantly lower (*p* < 0.001) compared to *Zygnema* sp. ‘Elmau-Alm’, while the opposite was found for the initial value of light-saturated photosynthesis (Table 2). Older cultures of *Zygnema* sp. ‘Saalach’ exhibited a continuously significant decrease (*p* < 0.001) in *rETR*_max_ (Fig. 6a). This was accompanied by decreasing *α* and *I*_k_ values; however, the culture with the highest age exhibited a similar *α* value like the youngest culture (Fig. 6a). In contrast, only the culture with the highest age of *Zygnema* sp. ‘Elmau-Alm’ showed a significantly decreased (*p* < 0.001) *rETR*_max_ value, while *I*_k_ values increased strongly with age (Fig. 6b). Older cultures always exhibited similar, however, significantly lower *α* values compared to the most recent culture of *Zygnema* sp. ‘Elmau-Alm’ (Fig. 6b).

**Table 2:** Characteristic parameters of rETR curves of the two *Zygnema* strains obtained from the most recent cultures on agar plates (~1 month)

Strain	*α*	*I*_k_	*rETR*_max_
*Zygnema* sp. ‘Saalach’	0.23 ± 0.03a	453.37 ± 112.75c	103.43 ± 13.22e
*Zygnema* sp. ‘Elmau-Alm’	0.31 ± 0.04b	146.74 ± 36.74d	61.74 ± 25.11f

Data were calculated by using the fitting model according to Webb et al. 1974 or Walsby 1997 depending on either photoinhibition occurred or not (*n* = 3, mean value ± SD). Different letters represent significant differences among the values. They were determined by a standard two-sample *t* test (*p* < 0.001)

*rETR*_*max*_ maximum electron transport rate (μmol electrons m^−2^ s^−1^), *α* initial slope at limiting photofluence rates (electrons photon^−1^), *I*_*k*_ initial value of light-saturated photosynthesis (μmol photons m^−2^ s^−1^)

### Photosynthetic oxygen production

Plotting photosynthetic oxygen production of the two *Zygnema* strains as a function of increasing photon fluence rates (PI curve) led to five characteristic photosynthesis parameters (Table 3) calculated with the fitting model of Webb et al. (1974), as no photoinhibition could be observed even under 500 μmol photons m^−2^ s^−1^. Photosynthetic rate in the light-saturated range (*P*_max_) was significantly higher (*p* < 0.001) in *Zygnema* sp. ‘Saalach’ compared to *Zygnema* sp. ‘Elmau-Alm’, while respiration amounted significantly higher in *Zygnema* sp. ‘Elmau-Alm’ (Table 3). Also, the values of alpha and *I*_k_ values differed significantly (*p* < 0.001). While the alpha value was conspicuously higher in *Zygnema* sp. ‘Elmau-Alm’, the *I*_k_ value was higher in *Zygnema* sp. ‘Saalach’ (Table 3). In contrast, the values of *I*_c_ showed no statistical difference between strains (*p* < 0.001; Table 3).

**Table 3:** Characteristic parameters of PI curves of the two *Zygnema* strains obtained from ~1-month-old liquid cultures

Strain	α	I_c_	I_k_	R	P_max_
*Zygnema* sp. ‘Saalach’	7.68 ± 2.40a	23.37 ± 6.20c	72.19 ± 34.35d	−152.56 ± 50.04f	527.20 ± 75.56h
*Zygnema* sp. ‘Elmau-Alm’	15.10 ± 0.74b	17.33 ± 0.72c	25.94 ± 1.45e	−195.20 ± 3.06g	390.67 ± 2.12i

Data were derived from PI curves using the fitting model according to Webb et al. 1974 (*n* = 3, mean value ± SD). Different letters represent significant differences among the values. They were determined by a standard two-sample *t* test (*p* < 0.001)

*α* initial slope in the light-limiting range (μmol O_2_ h^−1^ mg^−1^ chl. *a* (μmol photons^−1^ m^−2^ s^−1^), *I*_*c*_ light compensation point, *I*_*k*_ initial value of light-saturated photosynthesis (μmol photons m^−2^ s^−1^), *R* respiration rate in the dark (μmol O_2_ h^−1^ mg^−1^ chl. *a*), *P*_*max*_ maximum photosynthetic rate in the light-saturated range (μmol O_2_ h^−1^ mg^−1^ chl. *a*)

### Temperature requirements for photosynthesis

**Fig. 7:**
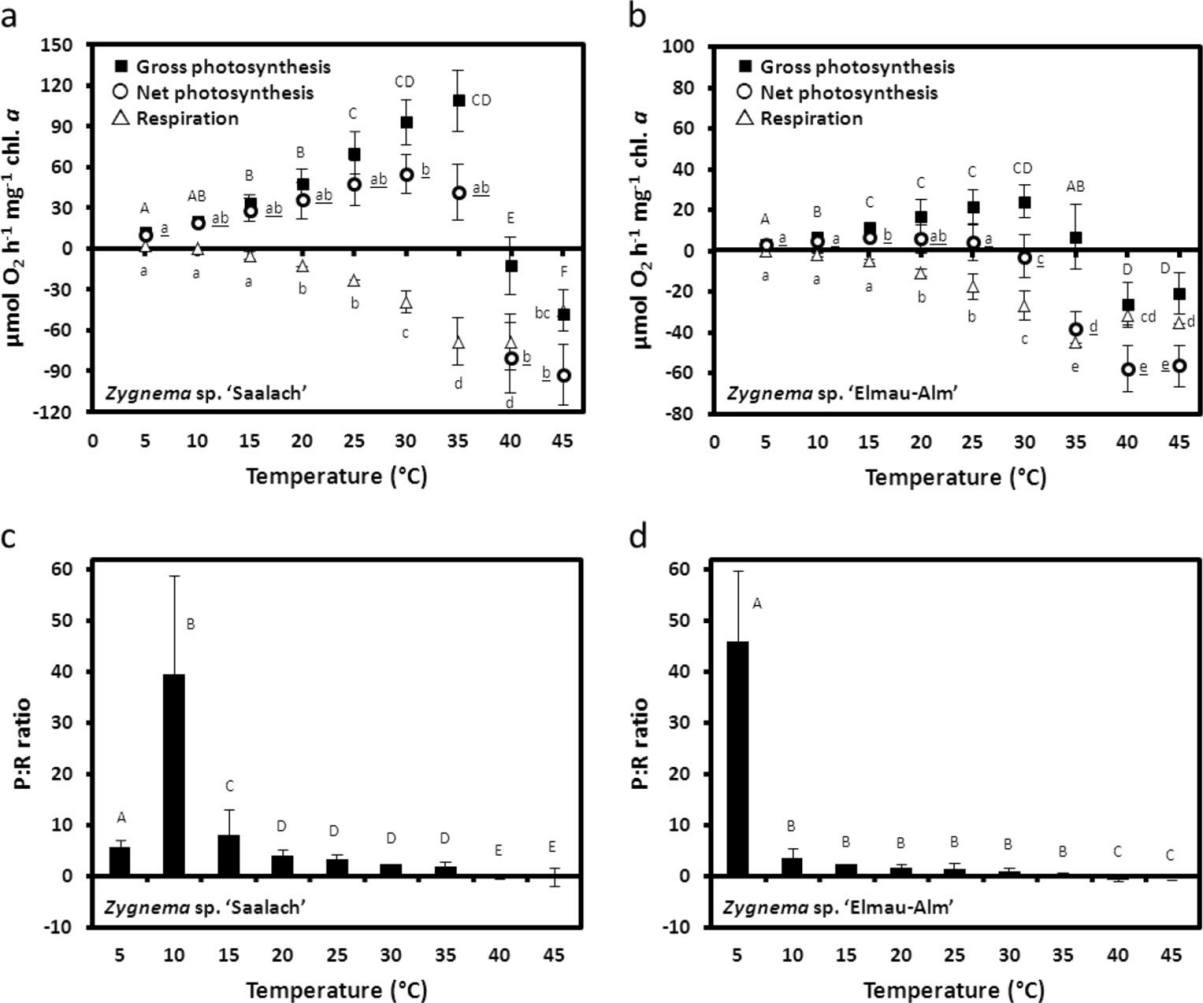
Gross and net photosynthetic oxygen production and respiratory oxygen consumption (μmol O_2_ h^−1^ mg^−1^ chl. *a*) in response to increasing temperatures in **a** *Zygnema* sp. ‘Saalach’ and **b** *Zygnema* sp. ‘Elmau-Alm’ (*n* = 3, mean value ± SD). Gross photosynthesis to respiration ratios (P/R) in response to increasing temperatures are shown for **c** *Zygnema* sp. ‘Saalach’ and **d** *Zygnema* sp. ‘Elmau-Alm’. Significant differences between the treatments are indicated by *capital letters* (gross photosynthesis, P/R), *underlined letters* (net photosynthesis) and *small letters* (respiration), as determined by one-way ANOVA (*p* < 0.05) followed by Tukey’s post hoc test. Please note a different scaling of the *y*-axis in **a** and **b**

Photosynthetic oxygen production as well as respiratory consumption exhibited strong temperature dependency; however, both physiological processes showed strong differences in sensitivity in response to increasing temperatures (Fig. 7). At 5 °C, photosynthesis in *Zygnema* sp. ‘Saalach’ amounted to 12.64 μmol O_2_ h^−1^ mg^−1^ chl. *a* (Fig. 7a). It increased almost linearly and reached its maximum at 35 °C (109.60 μmol O_2_ h^−1^ mg^−1^ chl. *a*; Fig. 7a), which corresponds to an 8-fold increase compared to the lowest temperature (Fig. 7a). Another increase of 5 °C resulted in a complete inhibition of photosynthetic oxygen production (−11.90 μmol O_2_ h^−1^ mg^−1^ chl. *a*; Fig. 7a). At 45 °C, oxygen evolution under lighting was almost identical to respiratory consumption (*P* = −47.81; *R* = −44–49 μmol O_2_ h^−1^ mg^−1^ chl. *a*; Fig. 7a). In contrast to photosynthesis, respiration was hardly detectable between 5 and 15 °C (0.61–4.86 μmol O_2_ h^−1^ mg^−1^ chl. *a*; Fig. 7a.). Respiratory oxygen consumption increased linearly to −67.8 μmol O_2_ h^−1^ mg^−1^ chl. *a* at 40 °C (Fig. 7a.). At 5 °C, the gross P/R ratio was 5.7 and showed a sharp increase at 10 °C followed by a drop to 7.8 at 15 °C and remained unchanged up to 35 °C (Fig. 7c). At 40–45 °C, no positive photosynthesis was detected (Fig. 7c).

Similar to *Zygnema* sp. ‘Saalach’, photosynthesis in *Zygnema* sp. ‘Elmau-Alm’ was very low at 5 °C (3.01 μmol O_2_ h^−1^ mg^−1^ chl. *a*) followed by an almost linear increase in response to increasing temperature (Fig. 7b). However, photosynthetic oxygen production reached its maximum already at 30 °C (23.60 μmol O_2_ h^−1^ mg^−1^ chl. *a*; Fig. 7b). At 35 °C, photosynthesis amounted to 6.48 μmol O_2_ h^−1^ mg^−1^ chl. *a*, while between 40–45 °C, no oxygen production could be observed (Fig. 7b). Respiratory oxygen consumption was almost not detectable between 5 and 15 °C; at 20 °C, a significant increase was measured followed by a linear increase up to 35 °C where respiration was −44.74 μmol O_2_ h^−1^ mg^−1^ chl. *a* (Fig. 7b). Increasing temperatures to 40 and 45 °C, respectively, caused decreasing respiration rates, however, being significantly higher than those at 30 °C (Fig. 7b). The P/R ratios in *Zygnema* sp. ‘Elmau-Alm’ showed a considerable 13-fold decrease from 5 to 10 °C followed by a smaller decrease from 30 to 35 °C (Fig. 7d). Again, at the highest temperature applied, the P/R ratio was negative due to absence of positive photosynthesis (Fig. 7d).

### Desiccation effects

**Fig. 8:**
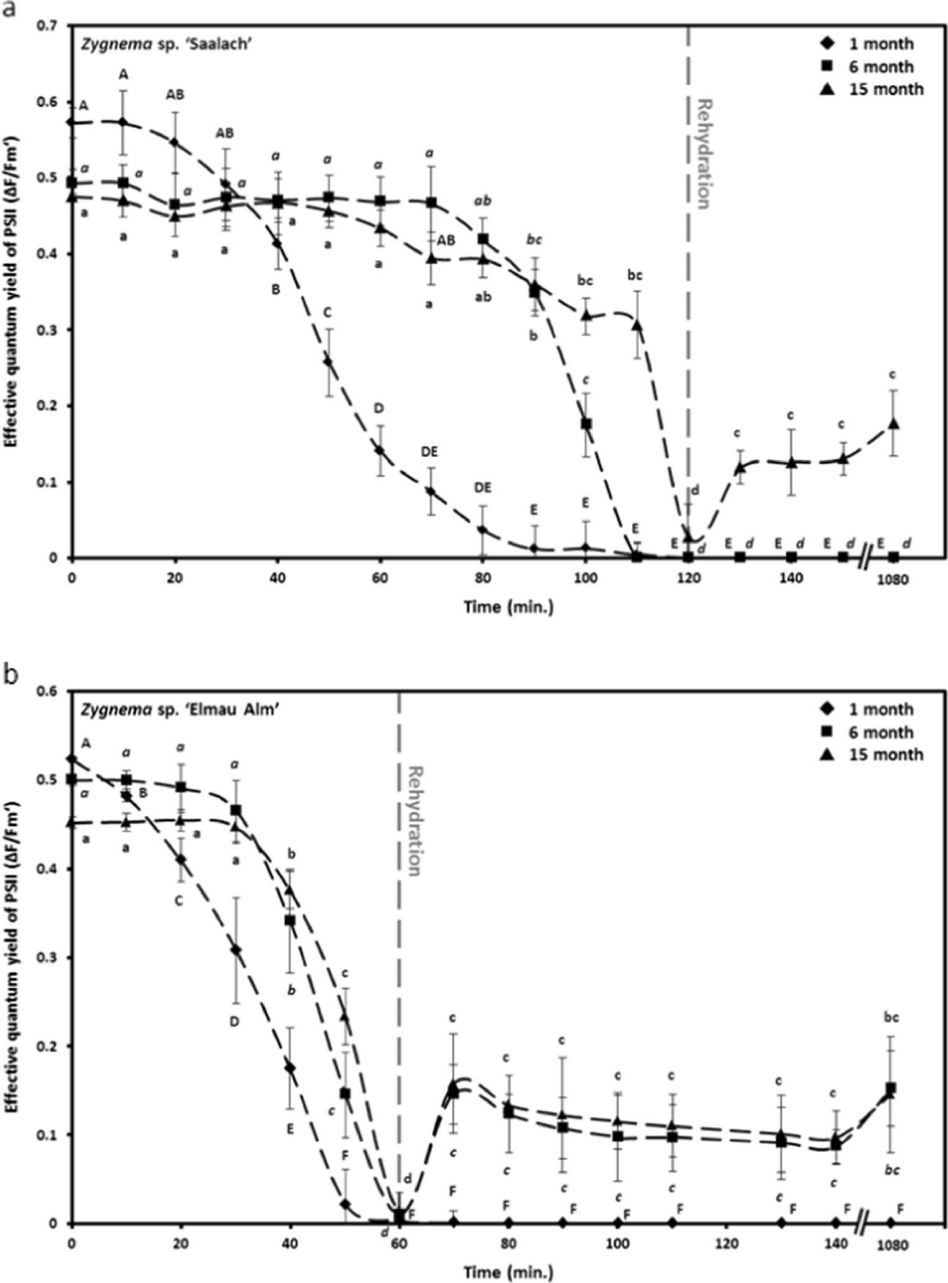
The effective quantum yield (Δ*F*/*Fm*′) of PSII in response to controlled desiccation, continuously measured in **a** *Zygnema* sp. ‘Saalach’ and **b** *Zygnema* sp. ‘Elmau-Alm’ (*n* = 4, mean value ± SD). Samples were taken from cultures of different culture age (1, 6 and 15 months). Rehydration for measuring recovery of Δ*F*/*Fm*′ followed after 120 min (*Zygnema* sp. ‘Saalach’) or 60 min (*Zygnema* sp. ‘Elmau-Alm’), respectively (*n* = 3, mean value ± SD). Δ*F*/*Fm*′ of control values was estimated under ~40 μmol photons m^−2^ s^−1^ PAR and determined in *Zygnema* sp. ‘Saalach’ as 0.57 ± 0.02 (*black diamond*), 0.49 ± 0.03 (*black square*) and 0.48 ± 0.02 (*black triangle*) and in *Zygnema* sp. ‘Elmau-Alm’ as 0.52 ± 0.03 (*black diamond*), 0.50 ± 0.01 (*black square*) and 0.45 ± 0.01(*black triangle*). Significances between the treatments are indicated by *capital letters* (*black diamond*), *cursive letters* (*black square*) and *small letters* (*black triangle*). They were determined by one-way ANOVA (*p* < 0.05) followed by Tukey’s post hoc test. Please note a different scaling of the *y*-axis in **a** and **b**

Comparing the effective quantum yield of the two *Zygnema* strains of different culture age during dehydration and rehydration revealed distinct strain- and age-specific differences (Fig. 8). After 10 min of desiccation, the youngest culture of *Zygnema* sp. ‘Saalach’ showed an almost linear decrease of Δ*F*/*Fm*′, reaching 0 after 90 min (Fig. 8a). In contrast, Δ*F*/*Fm*′ of 6- and 15-month-old cultures, which predominantly contained pre-akinetes, remained almost unchanged for over 70-min desiccation treatment, followed by a strong drop of fluorescence signal to 0 after 110 min (6 months) or 120 min (15 months), respectively (Fig. 8a). While the Δ*F*/*Fm*′ value of both the 1 and 6-month cultures stayed at a minimum after subsequent rehydration, the culture with the highest age partly recovered within 10 min (25.09 ± 11.26 % of initial value) and maintained that level after 18 h (Fig. 8a). The most recent culture of *Zygnema* sp. ‘Elmau-Alm’ showed a continuous decrease of Δ*F*/*Fm*′ in response to desiccation, reaching 0 after 50 min (Fig. 8b). The oldest cultures of *Zygnema* sp. ‘Elmau-Alm’ exhibited very similar kinetics during desiccation and subsequent rehydration; however, the initial Δ*F*/*Fm*′ value of the 6-month-old culture was significantly (analyzed by a standard two-sample *t* test, *p* < 0.05) higher (0.51 ± 0.01) compared to the 15-month-old culture (0.45 ± 0.01): Δ*F*/*Fm*′ dropped strongly after 30 min, reaching 0 after 60 min (Fig. 8b). Rehydration after 60 min was not followed by recovery of Δ*F*/*Fm*′ in the most recent culture (Fig. 8b). In contrast, both older cultures partly recovered within 10 min to 29.18 ± 21.60 % (6 months) or 34.87 ± 11.39 % (15 months) of the initial value, respectively, always followed by a slight increase after 18 h (Fig. 8b).

## Discussion

In the present study, a thorough characterization of two newly isolated *Zygnema* strains from alpine habitats was performed. We found that the strains were (1) phylogenetically distinct, belonging to different major subclades of the genus. The morphological and ultrastructural characterization demonstrated (2) differences between young and cultures of increasing age. (3) Significant differences in the photosynthetic performance of these two strains were measured and (4) an increase in desiccation tolerance was found in older cultures that contained predominantly pre-akinetes. We next discuss the phylogenetic position of the two strains, their cell structure and physiological performance in an ecological context, to contribute an understanding of early colonizing events of zygnematophycean green algae.

### Phylogenetic position

Within *Zygnema*, at least 137 species have been described mainly based on their reproductive morphology (Kadłubowska 1984). Differences in the zygospores resulting from the conjugation process, especially the colour and structure of the exospore and mesospore, are critical for species determination (Stancheva et al. 2012). Vegetative characteristics are less important for species identification within the Zygnematophyceae (Stancheva et al. 2012). For example, it has been shown for *Spirogyra* (Zygnematophyceae) that environmental conditions as well as polyploidy hybridization between species cause considerable vegetative morphological variation (McCourt et al. 1986; Stancheva et al. 2012). The occurrence of polyploidy is uncommon in the genus *Zygnema* (McCourt et al. 1986). However, it might not be fully excluded, as polyploidy has been found in several genera of the Zygnematophyceae (Hoshaw and McCourt 1988; Palamar-Mordvintseva et al. 1999; Poulíèková et al. 2014). Sexual reproduction was not observed in the *Zygnema* strains investigated in the present study; therefore, we performed phylogenetic analyses to facilitate assignment of these strains into the recent systematic treatment of *Zygnema* (Stancheva et al. 2012). The strains *Zygnema* sp. ‘Saalach’ and *Zygnema* sp. ‘Elmau-Alm’ were placed into different major subclades of the genus (Fig. 1). *Zygnema* sp. ‘Saalach’ was closest to several unnamed strains and *Z. circumcarinatum* and in the same subclade (but distantly related to) three *Zygnema* strains whose physiology was previously investigated (strains B, D and E). *Zygnema* sp. ‘Elmau-Alm’ was closest to a named strain (JH0453) and *Z. carinthiacum* from California, distantly related but in the same major subclade as the previously studied strain G, isolated from an Arctic habitat (Kaplan et al. 2013). 

### Morphology and ultrastructure

The two *Zygnema* isolates were obtained from alpine habitats of different altitudes (440 and 1,500 m a.s.l.) and were cultured for prolonged periods exceeding 1 year. Formation of pre-akinetes was induced in these older cultures. As light regime and temperature stayed unchanged during cultivation under hydrated conditions on agar plates and in liquid culture, nutrient attrition seems to be one key factor inducing formation of stationary phase cells in *Zygnema*. Nutrient starvation was also responsible for akinete formation in other taxa, e.g. Xanthophyceae (Darling et al. 1987; Nagao et al. 1999).

The structure of strains of *Zygnema* sp. (‘Saalach’ and ‘Elmau-Alm’) investigated was similar in appearance to *Zygnema* strains collected from different habitats, including the Arctic and Antarctic (e.g. McLean and Pessoney 1971; Holzinger et al. 2009; Pichrtová et al. 2013). As stated above, vegetative morphology, in general, has only limited significance for species identification in this genus. We found that *Zygnema* sp. ‘Saalach’ had markedly wider cells (23.2 μm) than *Zygnema* sp. ‘Elmau-Alm’ (18.7 μm). Therefore, it is particularly interesting that species from the same group as *Zygnema* sp. ‘Saalach’ (e.g. *Zygnema argillarii* Kadłub., *Zygnema irregular* H. Krieg. and *Zygnema giganteum* Randhawa; Stancheva et al. 2012) had wider cell diameters when compared to cells from the group of *Zygnema* sp. ‘Elmau-Alm’ (e.g. *Z. carinthiacum*, *Zygnema californicum* Stancheva, J.D. Hall and Sheath and *Zygnema aplanosporum* Stancheva, J.D. Hall and Sheath; Stancheva et al. 2012). Moreover, the cell width of *Zygnema* sp. ‘Saalach’ was found to be identical to the closely related *Z. circumcarinatum* (Miller and Hoshaw 1974). In contrast, *Zygnema* sp. ‘Elmau-Alm’ has smaller cells when compared to phylogenetically closely related species (e.g. *Zygnema peliosporum*; Stancheva et al. 2012). Thus, the different cell widths of the investigated strains correspond to their phylogenetic position. However, the cell diameter can also be influenced by, e.g., polygenic factors (Miller and Hoshaw 1974) or age. Especially in *Zygnema* sp. ‘Elmau-Alm’, decreasing cell width was strongly correlated with increasing culture age, and both strains had the smallest cell width in the oldest culture (15 months, Table 1). This corresponds to findings in *Mougeotia transeaui* Collins (Zygnematophyceae) which showed a decrease in cell width in clones established from 6-month-old cultures (Kennedy 1976). This varying cell width needs further investigation, as it was not the aim of the present study to investigate, if it is caused by either some physiological or some environmental (e.g. nutrient starvation) factors. The increasing cell length in 9 and 15 month (*Zygnema* sp. ‘Elmau-Alm’) or 15 month (*Zygnema* sp. ‘Elmau-Alm’) old cultures, respectively, might be explained by a decreasing cell division rate.

Both investigated *Zygnema* strains had a similar ultrastructual appearance to those from previous studies (e.g. McLean and Pessoney 1971; Bakker and Lokhorst 1987; Holzinger et al. 2009; Kaplan et al. 2013; Pichrtová et al. 2013). The cells possess two stellate chloroplasts. *Zygnema* ‘Saalach’ had only one pyrenoid per chloroplast, but *Zygnema* ‘Elmau-Alm’ occasionally had two. The centre of the pyrenoids had a characteristic shape with a central electron-dense area penetrated by thylakoid membranes, as observed by Pichrtová et al. (2013). The observed accumulation of starch grains allows concluding that the cells were very productive in culture, which is also corroborated by our photosynthesis measurements (see below). One common feature of older cells was large lipid bodies, as described previously for akinetes taken from 4 to 6-week-old cultures (McLean and Pessoney 1971). The earlier formation of akinetes observed by McLean and Pessoney 1971) might be explained by the use of different growth media. It was shown for *M. transeaui* (Zygnematophyceae) that filaments appeared to be healthier depending on chloroplast morphology when grown in BBM instead of Shen’s medium (Kennedy 1976), which was also used by McLean and Pessoney (1971). The induction of pre-akinete formation is likely linked to growth conditions. It is particularly interesting that under the same culture conditions, lipid bodies always accumulate in older cells, but they are virtually absent in young cells. While other authors describe little evidence for the occurrence of lipid bodies in cultivated *Zygnema* cells (Bakker and Lokhorst 1987), these studies likely did not investigate cultures which were aged to contain such cells. While lipid bodies were observed in pre-akinetes from both investigated strains in the present study, the electron-dense granules were mostly observed in *Zygnema* sp. ‘Saalach’. The later structures were particularly evident in field samples collected in Svalbard (Holzinger et al. 2009) as well as in cultivated Arctic and Antarctic samples (Pichrtová et al. 2013). Although their contents remain unknown, it is interesting that these structures have been found in close association to chloroplasts, suggesting a protective function. Perhaps they contain phenolic compounds, which were analyzed by HPLC in Arctic and Antarctic *Zygnema* sp. (Pichrtová et al. 2013).

### Photosynthetic oxygen production

In both strains, photosynthetic oxygen production and respiratory consumption as a function of increasing photon fluence rates recorded as PI curves show high *α* values in combination with low light compensation (*I*_c_) and saturation (*I*_k_) points. Although the *α* value, the positive slope at light-limiting photon fluence rates, was significantly higher and the *I*_k_ value significantly lower in *Zygnema* sp. ‘Elmau-Alm’ compared to *Zygnema* sp. ‘Saalach’, both strains can be considered as being adapted to low light conditions. Kaplan et al. (2013) reported similar values in four different *Zygnema* sp. strains from Arctic and Antarctic habitats. The two *Zygnema* strains investigated in our study had similar maximum photosynthetic oxygen production (*P*_max_) values when compared with the Arctic and Antarctic strains (Kaplan et al. 2013), pointing to a good photosynthetic performance. *Zygnema* sp. ‘Saalach’ exhibited a significantly higher *P*_max_ value compared to *Zygnema* sp. ‘Elmau-Alm’. Most interestingly, *Zygnema* sp. ‘Saalach’ is in the same subclade with strains previously shown to have high *P*_max_ values (Antarctic, *Zygnema* sp. E, D; Arctic, *Zygnema* sp. B, Kaplan et al. 2013). In contrast, *Zygnema* sp. ‘Elmau-Alm’ is related to the arctic *Zygnema* G, which showed the lowest *P*_max_ value in a previous study (Kaplan et al. 2013). This possible correlation between the photosynthetic performance and the phylogenetic position needs further investigation. This also indicates that green algae may have stable physiological characteristics even under long-term cultivation, as shown for several strains of the genus *Cosmarium* (Zygnematophyceae) whose photosynthetic behaviour appears to be genetically preserved and even unaffected after long-term cultivations for over 15 years (Stamenković and Hanelt 2013). Additionally, these *Cosmarium* isolates from different climatic zones showed ultrastructural characteristics that were consistent with their source location (Stamenković et al. 2014). Similar *P*_max_ values were also reported in *Ancylonema nordenskiöldii* Berggren (Zygnematophyceae) obtained from Arctic glaciers (*P*_max_ ~400 μmol O_2_ h^−1^ mg^−1^ chl. *a* at 20 °C; Remias et al. 2012). In *Zygogonium ericetorum* Kützing isolated from an Alpine streamlet at 2,350 m a.s.l., similar values for *P*_max_ were recorded (Herburger, unpublished observation). In *Mesotaenium berggrenii* (Wittrock) Lagerheim (Zygnematophyceae) occurring on bare glacier surfaces in alpine regions, *P*_max_ values were drastically lower (*P*_max_ ~200 μmol O_2_ h^−1^ mg^−1^ chl. *a* measured at 1 °C, reflecting natural temperature requirements; Remias et al. 2011). Additionally, the *I*_k_ and *I*_c_ derived from oxygen production curves measured in the present study were in the same range when compared to Arctic and Antarctic *Zygnema* strains (Kaplan et al. 2013). However, the light compensation point (*I*_c_) of *Zygnema* ‘Elmau-Alm’ was distinctly lower, which additionally points to a low light adaption of this strain. The significant low light adaption of *Zygnema* sp. ‘Elmau-Alm’ compared to *Zygnema* sp ‘Saalach’ is unanticipated, as one would expect higher solar radiation in their natural habitat, a sun exposed alpine pasture. The low light adaptation might be explained, in part, by a better photoprotection due to self-shading effects. *Zygnema* often forms multiple layers of filaments, and the upper layers shade deeper layers (Holzinger et al. 2009). This phenomenon has also been described in *Z. ericetorum* (Aigner et al. 2013).

*P*_max_ and respiration in Zygnematophyceae were higher when compared to other aeroterrestrial green algae, e.g. of the Klebsormidiophyceae (Karsten et al. 2010, 2013, 2014; Karsten and Holzinger 2012; Kaplan et al. 2012). However, these studies also showed clear low light adaption in Klebsormidiophyceae, which seems to be a common feature of aeroterrestrial green algae. As reported for most macroalgae, low light adaption of the photosynthetic apparatus is often related to strong photoinhibition under increasing photon fluence rates (e.g. Leukart and Hanelt 1995; Bischof et al. 1998; Holzinger et al. 2006). However, this was not the case in the two alpine *Zygnema* strains investigated, in different strains of *Interfilum* and *Klebsormidium* (Karsten et al. 2010, 2014; Karsten and Holzinger 2012; Kaplan et al. 2012), or in Arctic and Antarctic Zygnematophyceae (Kaplan et al. 2013; Remias et al. 2012). As earlier studies showed, however, such high photophysiological plasticity is not a common feature of all aeroterrestrial green algae, especially considering that algae occurring in dessert soil crusts were strongly photoinhibited under 130 μmol photons m^−2^ s^−1^ PAR (Gray et al. 2007). These algae may be well protected against intensive solar radiation due to their occurrence in soil crusts (Gray et al. 2007; Karsten and Holzinger 2014). 

### Relative electron transport rates and culture age

We performed rETR measurements with cultures of increasing age to test if young cells and pre-akinetes (see above) differ in another important photosynthetic parameter. In *Zygnema* sp. ‘Saalach’, the highest rETR values (*rETR*_max_) were measured in the youngest cultures lacking pre-akinete morphology (about 1 month after subculturing), which were also used for measurements of the photosynthetic oxygen production (see above). With increasing culture age (6, 9 and 15 months) accompanied by formation of pre-akinetes and akinetes, the *rETR*_max_ values decreased significantly. Only the culture with the highest age of *Zygnema* sp. ‘Saalach’ was photoinhibited under high light conditions, however, showing the same photosynthetic efficiency under low light conditions (*α* value) as the youngest cells. In contrast, the rETR in *Zygnema* sp. ‘Elmau-Alm’ was lower, and the curves appeared more similar across age, i.e. the young cells and the 6 and 9-month-old cells had a similar *rETR*_max_, with only the culture with the highest age having a significantly decreased *rETR*_max_. In this case, three of the four cultures were moderately photoinhibited but still showed positive rETR values even under 2,000 μmol photons m^−2^ s^−1^. This points to a variation in photophotoprotective functions between the two strains, e.g. the role of the xanthophyll cycle in non-photochemical quenching (Kranner and Birtic 2005; Lunch et al. 2013). In general, the here measured *rETR*_max_ values were substantially higher when compared to values obtained from Arctic and Antarctic strains (Kaplan et al. 2013). That said, Kaplan et al. (2013) occasionally used cultures for photosynthesis measurements that were up to ~10 weeks old, containing pre-akinetes. Photosynthetic efficiency can be expected to be slightly higher in younger cultures. In agreement with the photosynthetic oxygen production, *Zygnema* sp. G showed the lowest *rETR*_max_ value (Kaplan et al. 2013). As stated above, this strain is phylogenetically related to *Zygnema* sp. ‘Elmau-Alm’.

Comparatively, flattening of electron transport rate curves observed in older cultures of *Zygnema* sp. ‘Elmau-Alm’ was less strong, and a significant decrease of *rETR*_max_ was only observed in the oldest culture, while the 9-month-old culture was the only one lacking photoinhibition. Increasing age of cells of *Zygnema* sp. ‘Elmau-Alm’ seems to be accompanied by losing high plasticity in photophysiology, indicated by decreasing *α* values and increasing photoinhibition and *I*_k_ values. It is interesting that pre-akinetes of *Zygnema* still exhibit a good photosynthetic performance indicated by rETR, comparable to 3–5-week-old cultures lacking akinetes of other aeroterrestrial green algae (e.g. Klebsormidiophyceae; Karsten et al. 2010, 2014; Karsten and Holzinger 2012).

In both strains, maximum electron transport rates were reached under drastically higher photon fluence rates compared to maximum oxygen production; however, the multiplication factors between *rETR*_max_ and *P*_max_ were similar: *Zygnema* sp. ‘Saalach’, 6.3; *Zygnema* sp. ‘Elmau-Alm’, 5.7 (Tables 2 and 3). Under lower photon fluence rates (~0–100 μmol photons m^−2^ s^−1^), oxygen production increased strongly reaching a maximum (*P*_max_) at ~120 (*Zygnema* sp. ‘Elmau-Alm’) or ~170 μmol photons m^−2^ s^−1^ (*Zygnema* sp. ‘Saalach’), respectively. In contrast, under these light intensities, relative electron transport rates were still increasing in the about 1-month-old cultures, and an asymptote was reached under much higher photon fluence rates (1,000–1,300 μmol photons m^−2^ s^−1^). Similar findings were obtained from Arctic and Antarctic *Zygnema* sp. strains (Kaplan et al. 2013) and several marine macroalgae (Franklin and Badger 2001; Longstaff et al. 2002; Figueroa et al. 2003). This indicates an increase of electrons passing through PSII for every O_2_ produced (Longstaff et al. 2002). According to this, it is reasonable to assume additional sinks for transported electrons, when O_2_ production already reached a maximum. Possible sinks may be photorespiratory electron transport (Longstaff et al. 2002), cyclic electron flow around PSII (Falkowski et al. 1986) or the water-water cycle (Asada 2000). Evaluation of rETR, photosynthetic oxygen production and detection of Δ*F*/*Fm*′ (see below) at the beginning of desiccation treatment clearly indicated a higher photosynthetic efficiency of *Zygnema* sp. ‘Saalach’ compared to *Zygnema* sp. ‘Elmau-Alm’. 

### Temperature effects

Both respiratory oxygen consumption and photosynthetic production (see above) were strongly influenced by temperature (5–45 °C). In general, oxygen production was more effective under lower temperatures, whereas respiration was proportionally higher at higher temperatures. A comparison of the strains showed considerable differences in optima for photosynthesis and respiration. Optimum net photosynthesis in *Zygnema* sp. ‘Saalach’ was maintained up to 35 °C, while in *Zygnema* sp. ‘Elmau-Alm’, net photosynthesis was already strongly restricted at that temperature. Similarly, in *Zygnema* sp. ‘Saalach’, maximal respiration was maintained 5 °C above the maximum in *Zygnema* sp. ‘Elmau-Alm’. This is supported by the P/R ratios, pointing to a higher carbon gain in *Zygnema* sp. ‘Elmau-Alm’ under very low temperatures (5 °C) compared to *Zygnema* sp. ‘Saalach’, which exhibits the highest P/R ratio at 10 °C. In general, the P/R ratios indicate high net carbon gain under lower temperatures, indicating that biomass formation happens mainly under lower temperatures, which can be considered in agreement with temperature conditions in both habitats.

The temperature optima and the P/R ratios of the two strains are in good correlation with the different habitats from which they were isolated: *Zygnema* sp. ‘Saalach’ was isolated at 440 m a.s.l. in an environment exhibiting an annual mean temperature of 8.4 °C (mean temperature of the warmest month (August) = 17.9 °C; http://www.worldclimate.com). In contrast, *Zygnema* sp. ‘Elmau-Alm’ was isolated from a subalpine habitat (1,500 m a.s.l.), where significantly lower annual mean temperatures can be expected (Körner 2003). Theoretically, a decrease of 0.55 °C per 100 m is expected, which would result in a reduction of about 6 °C (Barry 2008). However, microclimate effects have to be considered as well (Körner 2007).

Different temperature requirements of photosynthesis and respiration, comparable to the two *Zygnema* strains investigated, have also been reported in other aeroterrestrial streptophyte green algae (e.g. *Interfilum* (Karsten et al. 2014) and *Klebsormidium* (Karsten et al. 2010; Karsten and Holzinger 2012)). *Ancylonema nordenskiöldii* isolated from Arctic glaciers also showed a significant increase in respiration and photosynthesis in response to higher temperatures. Biomass formation mainly occurred under lower temperatures, as increasing respiration reduces net carbon production at elevated temperatures (Remias et al. 2012). As discussed by Karsten et al. (2014), different temperature requirements for both physiological processes might be explained by lower temperature dependency of photosynthesis compared to respiration. The latter is composed of a chain of enzyme-controlled reactions with different temperature optima, taking place in various cellular compartments. Thus, the inhibition of only one process by low temperature can act as a bottleneck, restricting the whole process (Atkin and Tjoelker 2003).

### Desiccation effects

The experimental desiccation carried out in the present study was performed at a relative humidity of ~84 % over saturated KCl solution in a desiccation chamber (Karsten et al. 2014). We used cultures of different age to test if the formation of pre-akinetes is beneficial for desiccation tolerance, given that field populations of *Zygnema* frequently form these resistant cells (Pichrtová et al. 2014). Our findings clearly indicate that older cultures containing pre-akinetes are better protected against desiccation compared to young vegetative cells, lacking the obvious morphology of pre-akinetes. *Zygnema* sp. ‘Saalach’ tolerated 120 min of desiccation before the effective quantum yield (Δ*F*/*Fm*′) dropped to zero, which was used as an indicator for the photosynthetic performance. The Δ*F*/*Fm*′ values dropped more rapidly in the young culture, which reached zero after 90 min, and the 6-month-old culture took 110 min and the oldest culture (15 months) 120 min. In contrast, in *Zygnema* ‘Elmau-Alm’, Δ*F*/*Fm*′ dropped to zero at 60 min, and this was slightly earlier in the youngest culture.

Recovery to about ~30 % of the initial value of Δ*F*/*Fm*′ after subsequent rehydration was only found in the culture with the highest age of *Zygnema* sp. ‘Saalach’, but this was the case in both the 6 and 15-month-old cultures of *Zygnema* sp. ‘Elmau-Alm’. This clearly demonstrates that older cultures, predominantly containing pre-akinetes, have a greater resistance to desiccation, in terms of maintaining Δ*F*/*Fm*′, as well as better recovery rates upon rehydration.

The differences in desiccation tolerance observed may be related to the habitats where the algae were originally isolated (*Zygnema* sp. ‘Saalach’ occurred in the littoral zone of a river; *Zygnema* sp. ‘Elmau-Alm’ was isolated from shallow puddles), as well as to morphological differences such as cell width or the occurrence of mucilage. However, the most striking factor is the formation of pre-akinetes, clearly distinguishable by their ultrastructural appearance with accumulated lipid bodies and other storage compounds (see above). This involves thickening of cell walls, as well as accumulation of pectic layers (Fuller 2013) which might provide additional resistance to desiccation. It has been shown for several species of green algae that prolonged desiccation stress increases cell wall thickness (Morison and Sheath 1985; Bisson and Kirst 1995; Hoppert et al. 2004). In the present study, algal filaments were not pre-treated with desiccation prior to the experimental water stress over saturated salt solutions. A further factor in contributing to desiccation tolerance might be nutrient starvation as a consequence of prolonged cultivation (Pichrtová et al. 2014; Pichrtová, personal communication). However, we do not expect a drastic accumulation of osmolytes in older cells, as moist and wet field samples (Pichrtová et al. 2014) did not show different water potentials compared to liquid-culture-grown samples (Kaplan et al. 2013).

All of the findings obtained from laboratory experiments with stock cultures only pertain to short-term desiccation, but natural populations may behave distinctly different (Pichrtová et al. 2014). As hypothesized by Pichrtová et al. (2014), acclimation to the scarcity of naturally occurring water (e.g. slow desiccation or exposure to several desiccation-rehydration cycles) is necessary for the development of desiccation tolerance. It was found that wet field samples exhibited incipient plasmolysis at similar sorbitol concentrations as non-hardened cultures of *Zygnema* isolated from Arctic and Antarctic habitats, but dry collected samples were more resistant against osmotic stress (Kaplan et al. 2013; Pichrtová et al. 2014).

In contrast, *Klebsormidium crenulatum* (Kützing) H. Ettl & Gärtner isolated from alpine soil crusts, and air-dried for 3 h, resulted in a decrease of Δ*F*/*Fm*′ to under 20 % of the initial value, with full recovery after rehydration in only 2 h (Karsten et al. 2010). This ability could be attributed to a distinctly higher osmotic potential (*ψ* = −2.09 MPa) in *K. crenulatum* and resulting in a better water holding capacity (Kaplan et al. 2012). Four different strains of *Interfilum* were desiccated for more than 5 h at 10 % RH until Δ*F*/*Fm*′ decreased to zero (Karsten et al. 2014). Again, subsequent rehydration resulted in recovery of photosynthetic efficiency to 80–100 % of the initial value (Karsten et al. 2014), indicating a better recovery rate, compared to the strains currently investigated.

## Conclusion

We conclude that the two phylogenetically distinct *Zygnema* isolates are adapted to low light conditions, but are not strongly photoinhibited under high light conditions. Temperature regimes needed for positive net carbon gain are likely related to the different habitats from which the algae were obtained. With increasing culture age, a reduction of photosynthetic efficiency (rETR, *α* value and Δ*F*/*Fm*′) was observed, but older cells maintained Δ*F*/*Fm*′ longer when experimentally desiccated and showed better recovery rates. This is likely attributed to the formation of pre-akinetes in older cultures, which have been found to contain vast amounts of storage products. Therefore, cellular reorganization as a consequence of stresses such as nutrient depletion can contribute to the ability of *Zygnema* to survive unfavourable environmental conditions. Our findings illustrate the potential contribution of older cells in the successful colonization of terrestrial habitats. Desiccation tolerance in aeroterrestrial green algae needs further investigation as this is considered as one key factor for the transition of algae to terrestrial habitats (Becker and Marin 2009; Holzinger and Karsten 2013). Further studies should include genome sequencing approaches, as well as metabolomics and proteomics, for a deeper understanding of molecular mechanisms related to desiccation tolerance in green algae.

## References

[CR1] Aigner S, Remias D, Karsten U, Holzinger A (2013). Unusual phenolic compounds contribute to ecophysiological performance in the purple-colored green alga *Zygogonium ericetorum* (Zygnematophyceae, Streptophyta) from a high-alpine habitat. J Phycol.

[CR2] Altschul SF, Gish W, Miller W, Myers EW, Lipman DJ (1990). Basic local alignment search tool. J Mol Biol.

[CR3] Asada K (2000). The water–water cycle as alternative photon and electron sinks. Philos Trans R Soc Lond B Biol Sci.

[CR4] Atkin OK, Tjoelker MG (2003). Thermal acclimation and the dynamic response of plant respiration to temperature. Trends Plant Sci.

[CR5] Bakker ME, Lokhorst GM (1987). Ultrastructure of mitosis and cytokinesis in *Zygnema* sp. (Zygnematales, Chlorophyta). Protoplasma.

[CR6] Barry RG (2008). Mountain weather and climate.

[CR7] Becker B, Marin B (2009). Streptophyte algae and the origin of embryophytes. Ann Bot.

[CR8] Beniston M, Fox DG, Adhikary S, Andresson R, Guisan A, Holten JI, Ines J, Maitima J, Price M, Tessier L et al. (1996) The impacts of climate change on mountain regions. In: Second assessment report of the intergovernmental panel on climate change (IPCC). Cambridge University Press, Cambridge

[CR9] Billings WD (1973). Arctic and alpine vegetations: similarities, differences, and susceptibility to disturbance. Bioscience.

[CR10] Bischoff HW, Bild HC (1963). Phycological studies IV. Some soil algae from Enchanted Rock and related algal species. Univ Tex Publ.

[CR11] Bischof K, Hanelt D, Tug H, Karsten U, Brouwer PEM, Wiencke C (1998) Acclimation of brown algal photosynthesis to ultraviolet radiation in Arctic coastal waters (Spitsbergen, Norway). Polar Biol 20:388-395

[CR12] Bisson MA, Kirst GO (1995). Osmotic acclimation and turgor pressure regulation in algae. Naturwissenschaften.

[CR13] Coleman AW, Fryxell GA (1983). The roles of resting spores and akinetes in chlorophyte survival. Survival strategies of the algae.

[CR14] Darling RB, Friedmann EI, Broady PA (1987). *Heterococcus endolithicus* sp. nov. (Xanthophyceae) and other terrestrial *Heterococcus* species from Antarctica: morphological changes during life history and response to temperature. J Phycol.

[CR15] Davey MC (1991). The seasonal periodicity of algae on Antarctic fellfield soils. Holarct Ecol.

[CR16] Ellis EA (2006) Solutions to the problem of substitution of ERL 4221 for vinyl cyclohexene dioxide in Spurr low viscosity embedding formulations. Microsc Today 14:32–33

[CR17] Elster J, Benson EE, Fuller B, Lande N, Benson EE (2004). Life in the polar terrestrial environment: a focus on algae and cyanobacteria. Life in the Frozen State.

[CR18] Ettl H, Gärtner G (1995). Syllabus der Boden-, Luft- und Flechtenalgen.

[CR19] Evans JH (1958). The survival of freshwater algae during dry periods: part I. An investigation of the algae of five small ponds. J Ecol.

[CR20] Falkowski PG, Fujita Y, Ley A, Mauzerall D (1986). Evidence for cyclic electron flow around photosystem II in *Chlorella pyrenoidosa*. Plant Physiol.

[CR21] Figueroa FL, Conde-Alvarez R, Gomez I (2003). Relations between electron transport rates determined by pulse amplitude modulated chlorophyll fluorescence and oxygen evolution in macroalgae under different light conditions. Photosynth Res.

[CR22] Franklin LA, Badger MR (2001). A comparison of photosynthetic electron transport rates in macroalgae measured by pulse amplitude modulated chlorophyll fluorometry and mass spectrometry. J Phycol.

[CR23] Friedl T, Rybalka N (2012). Systematics of the green algae: a brief introduction to the current status. Progr Bot.

[CR24] Fritsch FE (1945). The structure and reproduction of the algae.

[CR25] Fuller CL (2013). Examining morphological and physiological changes in *Zygnema irregulare* during a desiccation and recovery period. Master’s thesis..

[CR26] Genty B, Briantais JM, Baker NR (1989). The relationship between the quantum yield of photosynthetic electron-transport and quenching of chlorophyll fluorescence. Biochim Biophys Acta.

[CR27] Gray DW, Lewis LA, Cardon ZG (2007). Photosynthetic recovery following desiccation of desert green algae (Chlorophyta) and their aquatic relatives. Plant Cell Environ.

[CR28] Greenspan L (1977). Humidity fixed points of binary saturated aqueous solutions. J Res Nat Bur Stand Sect A.

[CR29] Hall JD, McCourt RM, Delwiche CF (2008). Patterns of cell division in the filamentous Desmidiaceae. Close green algal relatives of land plants. Am J Bot.

[CR30] Hawes I (1989). Filamentous green algae in freshwater streams on Signy Island, Antarctica. Hydrobiologia.

[CR31] Hawes I (1990). Effects of freezing and thawing on a species of *Zygnema* (Chlorophyta) from the Antarctic. Phycologia.

[CR32] Holzinger A, Karsten U (2013) Desiccation stress and tolerance in green algae: consequences for ultrastructure, physiological, and molecular mechanisms. Front Plant Sci 4. doi: 10.3389/fpls.2013.00327 10.3389/fpls.2013.00327 PMC3749462 23986769

[CR33] Holzinger A, Karsten U, Lütz C, Wiencke C (2006). Ultrastructure and photosynthesis in the supralittoral green macroalga *Prasiola crispa* from Spitsbergen (Norway) under UV exposure. Phycologia.

[CR34] Holzinger A, Roleda M, Lütz C (2009). The vegetative arctic green alga *Zygnema* is insensitive to experimental UV exposure. Micron.

[CR35] Holzinger A, Lütz C, Karsten U (2011). Desiccation stress causes structural and ultra-structural alterations in the aeroterrestrial green alga *Klebsormidium crenulatum* (Klebsormidiophyceae, Streptophyta) isolated from an alpine soil crust. J Phycol.

[CR36] Hoppert M, Reimer R, Kemmling A, Schröder A, Günzl B, Heinken T (2004). Structure and reactivity of a biological soil crust from a xeric sandy soil in central Europe. Geomicrobiol J.

[CR37] Hoshaw RW, McCourt RM (1988). The Zygnemataceae (Chlorophyta): a twenty-year update of research. Phycologia.

[CR38] Huelsenbeck JP, Ronquist F (2001). MRBAYES: Bayesian inference of phylogeny. Bioinformatics.

[CR39] Kadłubowska J Z (1984) Conjugatophyceae I. Chlorophyta VIII. Zygnemales. In: Ettl H., Gerloff J, Heynig H, Mollenhauer D (eds): Süßwasserflora von Mitteleuropa. Band 16. G. Fischer, Stuttgart, Germany, p 1–532

[CR40] Kaplan F, Lewis LA, Wastian J, Holzinger A (2012). Plasmolysis effects and osmotic potential of two phylogenetically distinct alpine strains of *Klebsormidium* (Streptophyta). Protoplasma.

[CR41] Kaplan F, Lewis LA, Herburger K, Holzinger A (2013). Osmotic stress in the Arctic and Antarctic green alga *Zygnema* sp. (Zygnemtales, Streptophyta): effects on photosynthesis and ultrastructure. Micron.

[CR42] Karsten U, Holzinger A (2012). Light, temperature and desiccation effects on photosynthetic activity and drought-induced ultrastructural changes in the green alga *Klebsormidium dissectum* (Streptophyta) from a high alpine soil crust. Microb Ecol.

[CR43] Karsten U, Holzinger A (2014). Green algae in alpine biological soil crust communities: acclimation strategies against ultraviolet radiation and dehydration. Biodivers Conserv.

[CR44] Karsten U, Lütz C, Holzinger A (2010). Ecophysiological performance of the aeroterrestrial green alga *Klebsormidium crenulatum* (Klebsormidiophyceae, Streptophyta) isolated from an alpine soil crust with an emphasis on desiccation stress. J Phycol.

[CR45] Karsten U, Pröschold T, Mikhailyuk T, Holzinger A (2013). Photosynthetic performance of different genotypes of the green alga *Klebsormidium* sp. (Streptophyta) isolated from biological soils crusts of the Alps. Algol Stud.

[CR46] Karsten U, Herburger K, Holzinger A (2014). Dehydration, temperature and light tolerance in members of the aeroterrestrial green algal genus *Interfilum* (Streptophyta) from biogeographically different temperate soils. J Phycol.

[CR47] Kennedy FGR (1976) Biology of the green alga *Mougeotia transeaui* Collins. Dissertation, University of Arizona, pp 143

[CR48] Körner C (2003). Alpine plant life - functional plant ecology of high mountain ecosystems.

[CR49] Körner C (2007). The use of ‘altitude’ in ecological research. Trends Ecol Evol.

[CR50] Kranner I, Birtic F (2005). A modulation role for antioxidants in desiccation tolerance. Integr Comp Biol.

[CR51] Kromkamp JC, Forster RM (2003). The use of variable fluorescence measurements in aquatic ecosystems: differences between multiple and single turnover measuring protocols and suggested terminology. Eur J Phycol.

[CR52] Leliaert F, Smith DR, Moreau H, Herron MD, Verbruggen H, Delwiche CF, De Clerck O (2012). Phylogeny and molecular evolution of the green algae. Crit Rev Plant Sci.

[CR53] Leukart P, Hanelt D (1995) Light requirements for photosynthesis and growth in several macroalgae from a small soft-water stream in the Spessart Mountains, Germany. Phycologia 34:528-532

[CR54] Lewis LA, Seckbach J (2007). Chlorophyta on land: independent lineages of green eukaryotes from arid lands. Algae and cyanobacteria in extreme environments.

[CR55] Lewis LA, McCourt RM (2004). Green algae and the origin of land plants. Am J Bot.

[CR56] Lischke H, Guisan A, Fischlin A, Bugmann H, Cebon P, Dahinden U, Davies HC, Imboden D, Jaeger CC (1998). Vegetation response to climate change in the Alps: modeling studies. Views from the Alps: regional perspectives on climate change.

[CR57] Longstaff BJ, Kildea T, Runcie JW, Cheshire A, Dennison WC, Hurd C, Kana T, Raven JA, Larkum WD (2002). An in situ study of photosynthetic oxygen exchange and electron transport rate in marine macroalga *Ulva lactuca* (Chlorophyta). Photosynth Res.

[CR58] Lunch CK, LaFountain AM, Thomas S, Frank HA, Lewis LA, Cardon ZG (2013). The xanthophyll cycle and NPQ in diverse desert and aquatic green algae. Photosynth Res.

[CR59] McCourt RM, Hoshaw RW, Wang JC (1986). Distribution, morphological diversity and evidence for polyploidy in North American Zygnemataceae (Chlorophyta). J Phycol.

[CR60] McLean RJ, Pessoney GF (1970) A large scale quasi-crystalline lamellar lattice in chloroplasts of the green alga Zygnema. J Cell Biol 45:522–531 10.1083/jcb.45.3.522 PMC2107930 5459939

[CR61] McLean RJ, Pessoney GF, Parker BC, Brown RM (1971). Formation and resistance of akinetes of *Zygnema*. Contributions in phycology.

[CR62] McManus H, Lewis L (2011). Molecular phylogenetic relationships in the freshwater family Hydrodictyaceae (Sphaeropleales, Chlorophyceae), with an emphasis on *Pediastrum duplex*. J Phycol.

[CR63] Miller RD, Hoshaw RW (1974). Cell width as a taxonomic character with special reference to *Zygnema circumcarinatum* Czurda. Brit Phycol J.

[CR64] Morison MO, Sheath RG (1985). Responses to desiccation stress by *Klebsormidium rivulare* (Ulotrichales, Chlorophyta) from a Rhode Island stream. Phycologia.

[CR65] Nagao M, Arakawa K, Takezawa D, Yoshida S, Fujikawa S (1999). Akinete formation in *Tribonema bombycinum* Derbes et Solier (Xanthophyceae) in relation to freezing tolerance. J Plant Res.

[CR66] Palamar-Mordvintseva GM, Wasser SP, Nevo E (1999). On the flora of Zygnematales (Conjugatophyceae) of Israel. Int J Algae.

[CR67] Pichrtová M, Remias D, Lewis LA, Holzinger A (2013). Changes in phenolic compounds and cellular ultrastructure of arctic and Antarctic strains of *Zygnema* (Zygnematophyceae, Streptophyta) after exposure to experimentally enhanced UV to PAR ratio. Microb Ecol.

[CR68] Pichrtová M, Hajek T, Elster J (2014). Osmotic stress and recovery in field populations of *Zygnema* sp. (Zygnematophyceae, Streptophyta) on Svalbard (High Arctic) subjected to natural desiccation. FEMS Microbiol Ecol.

[CR69] Porra RJ, Thompson WA, Kriedmann PE (1989). Determination of accurate extinction coefficients and simultaneous equations for assaying chlorophylls a and b extracted with four different solvents: verification of the concentration of chlorophyll standards by atomic absorption spectroscopy. BBA–Bioenergetics.

[CR70] Poulíèková A, Mazalová P, Vašut RJ, Šarhanová P, Neustupa J, Škaloud P (2014). DNA content variation and its significance in the evolution of the genus *Micrasterias* (Desmidiales, Streptophyta). PLoS One.

[CR71] Remias D, Albert A, Lutz C (2010) Effects of simulated, but realistic, elevated UV irradiation on photosynthesis and pigment composition of the alpine snow alga *Chlamydomonas nivalis* and the Arctic soil alga *Tetracystis* sp. (Chlorophyceae). Photosynthetica 48:269-277

[CR72] Remias D, Schwaiger S, Aigner S, Leya T, Stuppner H, Lütz C (2011). Characterization of an UV- and VIS-absorbing, purpurogallin-derived secondary pigment new to algae and highly abundant in *Mesotaenium berggrenii* (Zygnematophyceae, Chlorophyta), an extremophyte living on glaciers. FEMS Microbiol Ecol.

[CR73] Remias D, Holzinger A, Aigner S, Lütz C (2012). Ecophysiology and ultrastructure of *Ancylonema nordenskiöldii* (Zygnematales, Streptophyta), causing brown ice on glaciers in Svalbard (high Arctic). Polar Biol.

[CR74] Ronquist F, Huelsenbeck JP (2003). MRBAYES 3: Bayesian phylogenetic inference under mixed models. Bioinformatics.

[CR75] Schreiber U, Bilger W (1993) Progress in chlorophyll fluorescence research: major developments during the past years in retrospect. Prog Bot 54:151–173

[CR76] Stamenković M, Hanelt D (2013). Protection strategies of *Cosmarium* strains (Zygnematophyceae, Streptophyta) isolated from various geographic regions against excessive photosynthetically active radiation. Photochem Photobiol.

[CR77] Stamenković M, Woelken E, Hanelt D (2014). Ultrastructure of *Cosmarium* strains (Zygenmatophyceae, Streptophyta) collected from various geographic locations shows species-specific differences both at optimal and stress temperatures. Protoplasma.

[CR78] Stancheva R, Sheath RG, Hall JD (2012). Systematics of the genus *Zygnema* (Zygnematophyceae, Charophyta) from Californian watersheds. J Phycol.

[CR79] Stancheva R, Hall JD, Herburger K, Lewis LA, McCourt RM, Sheath RG, Holzinger A (2014). Phylogenetic position and characterization of aplanospore formation in *Zygogonium ericetorum* Kütz. J Phycol.

[CR80] Swofford DL (2002). PAUP* version 4b10.

[CR81] Takahashi S, Murata N (2008). How do environmental stresses accelerate photo inhibition?. Trends Plant Sci.

[CR82] Timme RE, Bachvaroff TR, Delwiche CF (2012) Broad phylogenomic sampling and the sister lineage of land plants. PLoS one 7. doi: 10.1371/journal.pone.0029696 10.1371/journal.pone.0029696 PMC3258253 22253761

[CR83] Transeau E (1951). The Zygnemataceae.

[CR84] Vilumbrales DM, Skácelová K, Barták M (2013). Sensitivity of Antarctic freshwater algae to salt stress assessed by fast chlorophyll fluorescence transient. Czech Polar Rep.

[CR85] Walsby AE (1997). Numerical integration of phytoplankton photosynthesis through time and depth in a water column. New Phytol.

[CR86] Webb WL, Newton M, Starr D (1974). Carbon dioxide exchange of *Alnus rubra*: a mathematical model. Oecologia.

[CR87] Wodniok S, Brinkmann H, Glöckner G, Heidel AJ, Philippe H, Melkonian M, Becker B (2011). Origin of land plants: do conjugating green algae hold the key?. BMC Evol Biol.

[CR88] Zhao M, Running SW (2010) Drought-induced reduction in global terrestrial net primary production from 2000 through 2009. Science 329:940–943 10.1126/science.1192666 20724633

